# Sex-Based Differences in Management and Outcomes of Patients Admitted or Transferred to Advanced Therapy Centers for Heart Failure

**DOI:** 10.3390/jcm15072776

**Published:** 2026-04-07

**Authors:** Ilya Kim, Oluwatoba Akinleye, Jaya Kanduri, Pritha Subramanyam, Udhay Krishnan, Ilhwan Yeo, Jim Cheung, Luke Kim, Daniel Yang Lu

**Affiliations:** 1 Weill Cornell Cardiovascular Outcomes Research Group (CORG), Weill Cornell Medical College, New York, NY 10021, USA; 2 Department of Medicine, New York Medical College, Valhalla, NY 10595, USA; 3 Division of Cardiology, University of Minnesota Medical Center, Minneapolis, MN 55455, USA; 4 Cardiovascular Outcomes Research Group (CORG), Weill Cornell Medicine New York-Presbyterian Hospital, New York, NY 10021, USA

**Keywords:** advanced therapy center, invasive procedures, sex differences, inter-hospital transfer, outcomes

## Abstract

**Background:** Heart failure (HF) is a major public health challenge. Management at or transfer to advanced therapy centers (ATCs) is linked to greater procedural use and better outcomes for HF, however there is little data on the impact of patient sex on access to ATCs and transfer patterns. We evaluated sex-based differences in HF management and outcomes during admissions across center types and transfer status. **Method:** Adult HF admissions were identified in the 2016–19 Nationwide Readmissions Database. Centers performing ≥1 heart transplant or LVAD were classified as ATCs. Patients were stratified by sex and center type: (A) non-ATC admission, (B) ATC admission, (C) transfer to ATC. Multivariable regression adjusted for comorbidities and HF decompensations. **Results:** Among 2,872,268 weighted HF admissions (51.3% male), females were older, while males had more HF decompensations (cardiogenic shock, ventricular arrhythmias, mechanical ventilation, AKI). Females comprised only 39.6% of all transfers to ATCs (0.4% vs. 0.6%, OR 0.69, *p* < 0.001) and had a lower unadjusted mortality (2.6% vs. 2.8%, *p* < 0.001); however, rates of transfer and mortality were similar between sexes when adjusted for comorbidities and HF decompensations. Female patients were significantly less likely to receive invasive procedures (CRT/ICD, PCI, right heart catheterization, CABG, temporary mechanical support, ECMO, LVAD or heart transplant) across all hospital types and transfers. This disparity in procedural utilization persisted after multivariable adjustment and in sensitivity analysis of patients with severe HF. **Conclusions:** Females had lower frequency of transfer to ATCs. In-hospital mortality and transfer rates to ATCs were similar across patient sex when adjusted for comorbidities and HF decompensations. Females consistently underwent fewer diagnostic and therapeutic interventions across all center types and transfers.

## 1. Introduction

Heart failure (HF) remains one of the leading causes of hospitalization in the United States, with to over 1 million hospitalizations annually [1]. The prevalence of HF is over 5.7 million in the U.S. alone, with a projected 46% increase in prevalence to over 8 million adults and over $70 billion in direct healthcare costs by 2030 [2]. Despite advancements in guideline-directed medical therapies, remote cardiac monitoring, procedural interventions, and advanced therapies, HF continues have high morbidity and mortality [3]. Multiple factors likely contribute to these outcomes, including lack of timely access and referral for these therapies [4,5,6,7,8].

Many studies have demonstrated sex-based differences in the management and outcomes of HF [6,7,9,10,11]. These differences may stem from different underlying pathophysiology and comorbidity profiles as well as access to therapeutic interventions. Different studies have reported variable influence of sex on outcomes of HF across different clinical presentations [6,12,13,14,15,16]. Notably, among patients with cardiogenic shock, females have higher in-hospital mortality [17,18]. Females are less likely to undergo HF-related procedures, including revascularization, ICD implantation, Left Ventricular Assist Device (LVAD) implantation, or cardiac transplantation [6,7,9,10,18,19,20,21].

Our group previously demonstrated that tertiary hubs within hub-and-spoke networks capable of offering advanced therapies and other interventions or resources are associated with improved HF outcomes, and that patients transferred to these centers receive higher rates of diagnostic and interventional procedures corresponding with the improved outcomes [8]. However, there is little data on the influence of patient sex on access and transfer to these centers, receipt of advanced care and therapeutics, and outcomes of these HF hospitalizations.

The purpose of this analysis is to investigate sex differences in the characteristics, management, and outcomes of acute HF hospitalizations across admissions and transfers to different levels of care, using a contemporary, large, and nationally representative U.S. database.

## 2. Methodology

Data from the years 2016 to 2019 from the Nationwide Readmissions Database (NRD) were analyzed. The NRD is a publicly available, nationally representative database, maintained by the Agency for Healthcare Research and Quality (AHRQ), and derived from the Healthcare Cost and Utilization Project (HCUP) State Inpatient Databases. The NRD contains yearly discharge data for hospitalizations within multiple states, with unique patient linkage numbers to follow patients across multiple hospitalizations within a given year, and it contains diagnosis and procedure codes associated with each hospitalization, utilizing the International Classification of Diseases, Tenth Revision—Clinical Modification (ICD-10-CM) codes for the years studied. The 2019 NRD in particular was drawn from 30 HCUP States, accounting for 60.4% of all US hospitalizations, containing 18 million unweighted discharges and estimating approximately 35 million weighted discharges [22]. Institutional Review Board approval and informed consent were not required given all data were obtained from a deidentified database.

### 2.1. Advanced Therapy Centers

Advanced therapy centers (ATCs) were defined as hospitals that performed at least one Left Ventricular Assist Device (LVAD) implantation or heart transplant surgery in a given year. All other hospitals were defined as non-ATCs. LVAD implantation and heart transplantation were identified by the ICD-10-CM procedure codes listed in Table S1.

### 2.2. Inter-Hospital Transfers

Hospitalizations involving inter-hospital transfer were identified using the NRD variable SAMEDAYEVENT, while excluding transfers to rehab centers using the NRD variable REHABTRANSFER. In cases of inter-hospital transfer, the NRD combines admission records from each hospital involved in the transfer into a single record containing ICD-10-CM codes from both hospitals [22]. Only hospitalizations with a clear transfer or no-transfer designation were included in the analysis.

### 2.3. Study Population

Hospitalizations involving adults age ≥ 18 with the primary diagnosis code for the admission containing an ICD-10-CM code associated with HF (Table S1) were selected for the analysis. The index admission was defined as the first HF admission for each patient in a given year. Index admissions from the last 90 days of the year were excluded to allow for 90-day readmission analysis.

Hospitalizations were stratified by sex and compared among 3 cohorts: (A) direct admissions to non-ATC (no transfer), (B) direct admissions to ATC (no transfer), and (C) transfers to ATC. A combination of ICD-10-CM codes (Table S1), NRD variables, and AHRQ comorbidity measures were used to characterize sociodemographic characteristics, comorbidities, etiologies of HF, outcomes, utilization of HF-related procedures, and readmissions. Six major HF decompensations were identified to compare and adjust for severity of HF admissions: cardiogenic shock, cardiac arrest/cardiopulmonary resuscitation, mechanical ventilation/intubation, non-invasive ventilation > 24 h, ventricular arrhythmias, and acute kidney injury (AKI). Readmission analysis included only patients who survived their index admission.

### 2.4. Statistical Analysis

Analyses were performed using SAS, version 9.4 (SAS Institute, Cary, NC, USA). All analysis accounted for complex survey design utilizing survey-specific statements (SURVEYFREQ, SURVEYMEANS, SURVEYLOGISTIC) and accounted for hospital-level clustering of patients and sampling design using the CLUSTER and STRATA statements, respectively, in accordance with AHRQ recommendations [23]. Survey-specific discharge weights provided by the NRD were used to calculate nationally representative estimates. The Rao–Scott χ^2^ test was used to compare categorical variables, and the Mann–Whitney–Wilcoxon nonparametric test was used for continuous variables. Multivariable logistic regression models were used to evaluate the association between the sex and study outcomes, adjusting for clinically pertinent comorbidities, sociodemographic factors, and the above-listed HF decompensations. Cost analysis was performed using cost-to-charge ratios provided by HCUP. All statistical tests were 2-sided, with *p* < 0.01 denoting statistical significance.

### 2.5. High-Volume ATCs

As a sensitivity analysis, the primary analysis was repeated utilizing a more stringent criteria for high-volume ATCs, defined as hospitals within the top tertile of LVAD or heart transplant annual procedural volume. Patients were categorized as direct admissions to non-high-volume ATCs, direct admissions to high-volume ATCs, or transfers to high-volume ATCs.

## 3. Results

Out of a total of 2,872,268 index HF hospitalizations from 2016 to 2019, 51.3% of patients were male and 48.7% were female (Table 1). A total of 81.2% were directly admitted to non-ATCs (Group A), 18.3% were directly admitted to ATCs (Group B), and 0.5% were transferred to ATCs (Table 2). ATCs comprised 5.5% of all centers.

### 3.1. Baseline Characteristics

Characteristics and comorbidities of males and females admitted with HF are shown in Table 1. Females were generally older (median 76 vs. 71 years, *p* < 0.001), with over 40% of all females above 80 years of age. Females had a higher rate of Medicare as primary insurance, likely reflecting older age, while males had higher rates of private insurance and self-pay/other. Comorbidities varied significantly by sex. Males had more coronary artery disease, CKD, liver disease, coagulopathy, and smoking/alcohol/substance use. Females had more valvular disease, thyroid disorders, autoimmune conditions, and depression. Males generally had higher rates of HF decompensations, including cardiogenic shock, ventricular arrythmias, and AKI.

Table 2 shows baseline characteristics stratified by both patient sex and hospitalization group. Within all three hospitalization cohorts, females were older than males, and the different patterns of sex-related comorbidities were generally similar to Table 1. Both males and females who were admitted to ATCs had significantly higher rates of HF decompensations compared to non-ATCs, especially transferred patients. Males transferred to ATCs had particularly higher rates of HF decompensations and generally greater than females, including cardiogenic shock (M: 37.6%, F: 26.5%, *p* < 0.001), ventricular arrhythmias, (M: 31.4%, F: 17.0%, *p* < 0.001), and AKI (M: 63.5%, F: 51.6%, *p* < 0.001); each of these decompensation rates were much higher than non-transferred patients at either hospital type. The rate of MI was slightly lower in non-transferred female patients; in transferred patients MI was much more frequent overall but not significantly different between sexes. Females were overall less likely to be transferred to an ATC compared to males (M: 0.6%, F: 0.4%, OR 0.69, *p* < 0.001) and comprised only 39.6% of all ATC transfers.

### 3.2. Hospitalization Outcomes

Females had slightly lower overall in-hospital mortality than males (2.6% vs. 2.8%, OR 0.94, *p* < 0.001) (Table 3 and Table 4). Lengths of stay and costs were numerically similar between sexes in the non-transferred cohorts but higher for males who were transferred (group C) compared to females. The number of 90-day readmissions was similar between sexes.

After multivariable adjustment for demographics, comorbidities, and severity of HF as represented by HF decompensations, there were no sex-related differences for in-hospital mortality (OR 0.99, *p* = 0.404) (Table 4) or rate of transfer to ATCs (OR 1.05, *p* = 0.097) (Table S2C). ATCs were associated with improved adjusted mortality for both transferred and non-transferred patients (Table S2A) as previously reported [8]. For transfers, predictors of transfer notably included younger age, better socioeconomic status (private insurance, higher income quartile), MI, valvular disease, and most HF decompensations including cardiogenic shock and ventricular arrhythmias (Table S2C). In every hospitalization cohort, females were less likely to be discharged home and more likely to be discharged to a facility (Table 2).

### 3.3. Procedural Utilization

**Figure 1:**
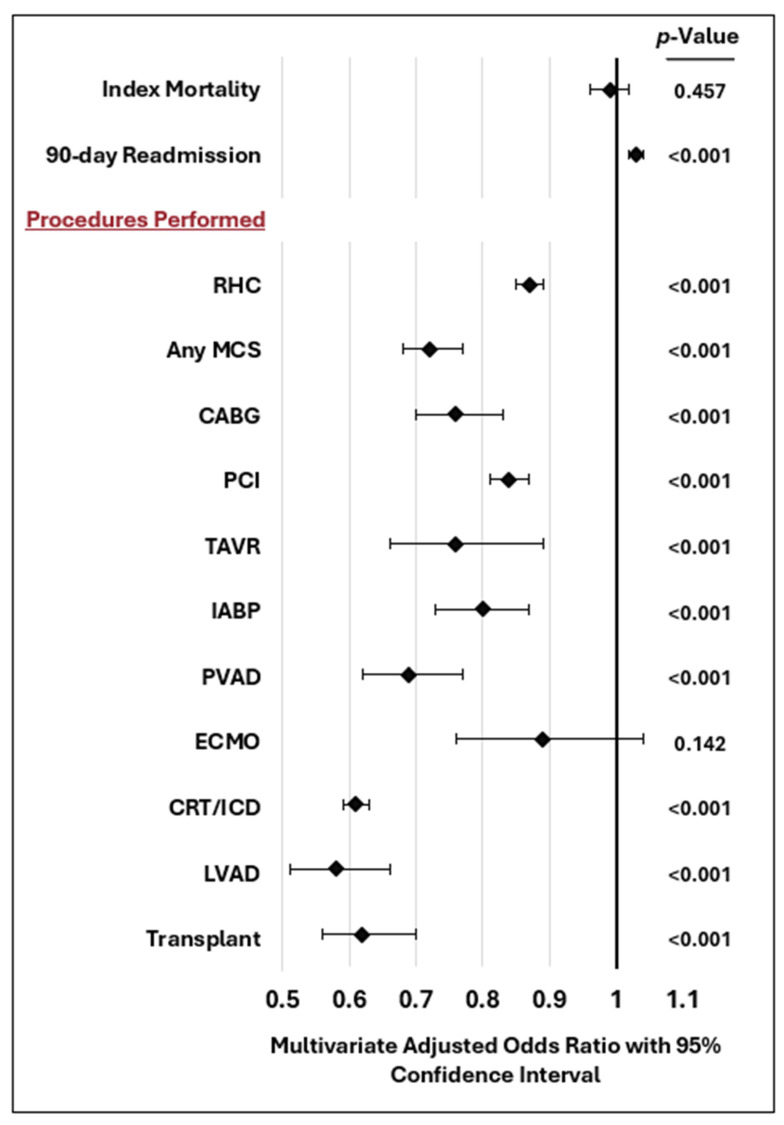
Multivariable Analysis of the Association of Female Sex with Heart-Failure Management and Outcomes. Abbreviations: RHC: Right Heart Catheterization; PCI: Percutaneous Coronary Intervention; MCS: Mechanical Circulatory Support; CABG: Coronary Artery Bypass Grafting; TAVR: Transcatheter Aortic Valve Replacement; IABP: Intra-Aortic Balloon Pump; PVAD: Percutaneous Ventricular Assist Device; ECMO: Extracorporeal Membrane Oxygenation; CRT/ICD: Cardiac Resynchronization Therapy/Implantable Cardioverter-Defibrillator; LVAD: Left Ventricular Assist Device; ATC: Advanced Therapy Center; LVADs and heart transplants were not performed in non-ATCs.

Procedure utilization was generally higher at ATCs, and especially high among transferred patients (Table 3, Figure S1). Between sexes, females underwent significantly fewer invasive procedures than males in every hospitalization cohort. Even after adjusting for comorbidities and severity of HF as represented by decompensations, females remained significantly less likely to receive HF-related procedures, including revascularization, CRT/ICD, right heart catheterization (OR 0.87, *p* < 0.001, Table S3), temporary mechanical support (OR 0.72, *p* < 0.001, Table S3), or advanced therapies including LVAD and heart transplantation (Table 4, Figure 1). A sensitivity analysis that included procedural use as variables in the multivariable analysis suggested that while various MCSs were associated with increased mortality, other procedures including right heart catheterization, revascularization, CRT/ICD, and LVAD/transplant were associated with lower mortality (Table S2B). Findings were similar in another sensitivity analysis restricting the definition of ATCs to only high-volume LVAD and transplant centers (top tertile of annual volume), with similar observed patterns of mortality, transfer, and procedural use with respect to patient sex (Tables S4 and S5). The results of interaction testing are listed in Table S9.

### 3.4. Heart-Failure Etiology

Table 5 shows significant sex-based differences in the classification of HF etiology based on ICD-10 codes associated with each hospitalization. Both sexes had high rates of ICD-10 codes associated with hypertensive HF. Males had higher prevalence of ischemic cardiomyopathy, dilated cardiomyopathy, alcoholic cardiomyopathy, and drug-induced cardiomyopathy. Females were more frequently diagnosed with hypertrophic cardiomyopathy, Takotsubo cardiomyopathy, peripartum cardiomyopathy, and heart failure with preserved ejection fraction (HFpEF).

### 3.5. Severe Heart Failure

A sub-analysis was performed with a subset of more severe HF patients as defined by presence of the three highest risks for mortality—cardiogenic shock, cardiac arrest, or mechanical ventilation during the index admission (Tables S2A, S6 and S7). There were 146,156 patients with severe HF on index admission, with males comprising a majority of this population (61.3% male, 38.7% female), and 7825 (5.4%) of these patients were transfers to ATC (Table S6). Unadjusted mortality rate was higher among female patients (34.4% vs. 31.4%, *p* < 0.001), but there was again no significant difference in in-hospital mortality between sexes on multivariable analysis (Table S8). Even in this severe HF population, female sex again predicted lower rates of right heart catheterization, mechanical circulatory support, and advanced therapies including LVAD and heart transplant, but there was no difference in coronary revascularization and CRT/ICD (Table S8).

## 4. Discussion

To our knowledge, this is the largest contemporary study of sex-based differences in HF hospitalizations across higher and lower level care centers and the first large study to examine the influence of patient sex on inter-hospital transfers. We highlight the following key findings: (1) females were less likely to be transferred to advanced therapy centers and had slightly lower mortality, but transfer rates and mortality when adjusted for comorbidities and decompensations were not different between males and females, (2) male and female HF patients have different comorbidity profiles, with males having higher severity of illness as measured by HF decompensations, (3) females are less likely to receive important diagnostic or therapeutic procedures, even if admitted or transferred to an ATC, and (4) the patterns of HF etiologies were different between males and females.

Our study found significant sex-based differences in phenotypic and comorbidity profiles. Males had more ischemic disease, higher rates of kidney and liver disease, and higher rates of substance use. Females were generally older, and more likely to present with valvular pathologies, autoimmune conditions, thyroid conditions, and depression. Regarding HF etiologies, males were more likely to present with ischemic, dilated, and alcohol-/drug-related cardiomyopathies, and females were more likely to present with nonischemic and non-substance-related etiologies, such as hypertrophic, Takotsubo, peripartum cardiomyopathies, as well as HFpEF. The sex-based differences in demographic and comorbidity profiles identified in our study may contribute to the outcomes seen (especially relating to procedure utilization and transfer), and align with patterns previously characterized in the literature, with females being older at the time of HF diagnosis, having higher rates of nonischemic etiologies, and more autoimmune and thyroid disease [9,24]. The prior literature has also shown that females are more likely to have HFpEF, consistent with our study [6,9,10,15,25].

HF admissions can range widely in severity, from mild HF requiring a few doses of IV diuretics to severe decompensated HF. Importantly, males in our study were more likely to be sicker during hospitalization, as measured by the co-existing presence of HF-related decompensations, such as mechanical ventilation, cardiac arrest, cardiogenic shock, AKI, or ventricular arrhythmias. These trends were consistent both at non-ATCs and ATCs, including patients transferred to an ATC. These results are similar to a prior study in the Nationwide Inpatient Sample (NIS) which showed that females hospitalized with HF had lower incidence of mechanical ventilation, vasopressor use, or mechanical circulatory support compared to males [12].

In our study, females had slightly lower overall in-hospital mortality. Sex-specific differences in in-hospital mortality for HF vary in the literature. Multiple studies and reviews of large patient registries have shown lower in-hospital mortality among females admitted with acute HF [12,26]. Multiple factors have been implicated in the lower observed mortality among females, including different comorbidity and HF etiology profiles (higher ejection fraction, lower ischemic disease, higher systolic pressure), and favorable physiological mechanisms and myocardial adaptation to stress [12,27]. On the other hand, reviews of other outcomes from randomized trials and national/international registries found no mortality difference between males and females hospitalized with HF [9,10,11,14,15,16,28]. Within our study, males tended to have higher rates of HF decompensations, and these decompensations each conferred several-fold increases in mortality risk, with the exception of ventricular arrhythmias—suggesting that the higher mortality among males can be explained by the sicker population of males on presentation. There was no difference between sexes in mortality after multivariable adjustment which included HF decompensations, comorbidities, and center type. Differences in etiology of HF may also play a role, such as lower prevalence of ischemic cardiomyopathy and substance-related cardiomyopathy, and higher prevalence of HFpEF, peripartum cardiomyopathy, and Takotsubo cardiomyopathy in females—which are associated with different clinical trajectories and prognoses.

It is possible that inconsistent sex-specific differences in mortality among HF admissions across various studies may reflect differences in patterns of care utilization. Interaction between access to care and mortality was suggested in the global REPORT-HF registry, which showed that females hospitalized with HF have lower in-hospital mortality only in countries with low income disparity, while in nations with higher income disparity this mortality advantage was not seen [6]. Although females with HF may benefit from more favorable biological mechanisms, differences in treatment patterns or healthcare delivery may affect outcomes compared to males [27].

The prior literature has described differences in care utilization between males and females with heart failure. For example, with regard to specialized care, females are less likely to be treated at a specialized cardiology unit as opposed to a general medicine unit, and less often referred to specialized HF clinics after an ED visit with a HF diagnosis [5,15]. Females with HF are less likely to receive optimal management, with less-intensive diuresis and lower overall weight loss during hospitalization, and are less likely to be prescribed guideline-directed medical therapy [9,11,29,30]. In the ADHERE registry, females had less weight loss during hospitalization, were less likely to undergo vasoactive therapy and invasive procedures, including CABG, cardioversion, right heart catheterization, pacemaker implantation, or transplant [10].

To our knowledge, there is little data describing sex-based differences in transfer patterns to ATCs among patients admitted for HF. In our study, ATCs were defined as centers who have performed at least 1 LVAD or heart transplant, reflecting a center among the highest levels of capability, resources, institutional infrastructure, and accreditation in treating heart failure. Within “hub-and-spoke” models of heart failure care, ATCs serve as HF hubs and are considered the gold standard for HF treatment as well as many other aspects of cardiology care, providing not only advanced therapies such as LVAD or transplant, but also expertise in multiple cardiac subspecialties and conditions, such as HF, cardiogenic shock, MI, and arrythmias [8,31]. ATCs offer procedural availability and proficiency (such as PCI, mechanical circulatory support, electrophysiology procedures, cardiac and non-cardiac surgeries), along with nonprocedural resources such as dedicated cardiac care units, equipment, nursing ratios, and training/expertise of nursing and allied health professionals [8,32]. Patients treated at ATCs have improved outcomes, both for directly admitted and transferred patients [8].

In our study, females were significantly less likely to be transferred to ATC (OR 0.69), comprising only 40% of all transfers. However, transfer rates between sexes were similar after multivariable adjustment for comorbidities and HF decompensations. This suggests that one possible reason for the difference in transfer rates is the higher severity of HF admissions in males and differences in comorbidity prevalence, including older age in females. Additionally, in real world clinical practice, transfers can often occur with targeted procedural intervention in mind, including revascularization, advanced therapies, or mechanical circulatory support. In our cohort, transferred patients have substantially higher rates of procedural intervention compared to the other two cohorts. It is possible that lower rates of procedural utilization in females (seen across all three cohorts) may also be associated with the observed sex-based differences in transfer patterns. However, the limitations of the dataset (including lack of granular details such as hemodynamics, patient/provider preference, goals of care, socioeconomic status, eligibility for advanced therapies and procedures) do not allow us to draw any definitive conclusions regarding intent or reason precluding or surrounding transfer. Further prospective research is needed to better understand clinical decision-making, candidacy, the geographical and socioeconomic factors, and timing surrounding transfer for females with HF, and to better understand if prospectively optimizing transfer pathways can further improve outcomes.

Our study shows that females are less likely to be discharged home and more likely discharged to a facility. This is consistent with other national studies, and in part explained by greater frailty associated with older age, higher prevalence of weight loss and malnutrition, depression and other comorbidities [12,33].

In our study, females across all three cohorts had lower procedural utilization across a range of HF-related diagnostic and therapeutic procedures (including right heart catheterization, coronary revascularization, mechanical circulatory support, CRT/ICD, and advanced therapies). While patients at ATCs, especially those transferred, were generally more likely to receive invasive procedures—females still consistently had lower rates of procedural utilization compared to males regardless of hospital type or transfer status. While the limitations of the dataset do not fully allow elucidation of the reasons behind the lower procedural utilization, it is possible that part of this observed sex-related difference may be related to differences in age, comorbidity burden, HF etiologies, or illness severity, as multivariable analysis reduced the magnitude of the differences in procedural use. For example, females were generally older in our study, and advanced age or greater frailty may influence clinical decision-making regarding invasive procedures. However, unlike transfer rates, even after multivariable adjustment for these factors such as for age, MI, and cardiogenic shock, the sex-related differences in procedural utilization remained significant. Furthermore, a second analysis including only the sickest patients (with cardiogenic shock, mechanical ventilation, or cardiac arrest) continued to demonstrate lower procedural use in females.

Our data is consistent with the prior literature, as lower procedural use in females has been reported across a variety of cardiovascular conditions, including HF, cardiogenic shock, and MI [6,10,18]. A review of 4.5 million HF patients in Germany showed lower utilization of PCI and ICD in females [7]. While ICD implantation is underutilized among all HF patients, females have comparatively lower rates of referral and ICD implantation rates [19,34,35,36,37]. Females are also less likely to undergo right heart catheterization and mechanical circulatory support and receive proportionally fewer heart transplants and LVADs [18,20,21].

Multiple factors have been proposed in the literature to explain the observed sex-related differences, including differences in recognition of cardiovascular symptoms among females, potential implicit bias among care providers in clinical assessment, or differences in symptom reporting patterns which may lead to delayed diagnosis and therapy [38,39,40]. Additionally, there are limited recommendations by guidelines due to inadequate robust clinical trial data on sex-specific differences in HF [1,41,42]. Socioeconomic disparities between males and females may affect access to care [6]. Finally, procedural complication rates have been shown to be higher in females, including vascular complications, bleeding, and kidney injury, which may influence clinical decision-making regarding invasive procedures, even when they may otherwise be indicated [43,44,45,46]. These observations underscore the need for further prospective research to continue to clarify sex differences in clinical utilization in heart failure, identify avenues to improve outcomes, and help further inform future research and guideline development.

Our study benefits from a large, nationally representative, real-world sample of HF hospitalizations, with the ability to identify hospitals by level of care and classify transfer status. Several limitations also apply. First, our study is retrospective and observational, utilizing an administrative database based on diagnosis codes, thus subject to coding inaccuracies. Specifically, HF etiologies may often be complex and overlapping and may be misclassified on initial presentation just based on ICD-10 codes. However, HCUP performs routine quality control of NRD to verify validity and reliability [47]. Second, the database lacks detailed clinical information important for clinical decision-making, especially as they relate to decisions on transfer and procedural use, such as lab values of end-organ function, echocardiographic findings such as ejection fraction or severity of valvular abnormalities, Intermacs/SCAI stages, perfusion or hemodynamic parameters, functional status, anatomy, socioeconomic factors, patient and provider preference, and medication regimen and adherence. The NRD also lacks detailed patient characteristics that are important in the context of LVAD candidacy or transplant allocation, including blood type, height, weight, body surface area, and vascular access size. These factors and their impact on transfers and procedural utilization deserve further attention in future prospective studies. Third, the NRD does not include detailed geographic identifiers such as distance or region, limiting the assessment of regional referral and transfer patterns between hospitals. Further research will be needed to evaluate geographical impact given its potential importance in influencing transfer decision-making. Fourth, because NRD is derived from separate and distinct state inpatient databases, a small portion of transfers may go unrecognized, particularly those occurring across state lines or to and from federal hospitals. NRD also does not specify the transfer origin, thus a small portion of transfers may represent transfers between ATCs. Fifth, since the NRD links hospitalizations involving transfer into a single encounter, it is unable to determine the timing of procedures, decompensations, or other clinical events in relation to transfer, further limiting understanding of clinical decision-making surrounding transfer. Sixth, the NRD lacks data on race/ethnicity, which can interact with sex on clinical outcomes. Finally, the NRD does not differentiate sex, gender, and gender identity, therefore the analysis in the study reflects sex as recorded in administrative data and cannot account for gender-related social or behavioral factors.

## 5. Conclusions

To our knowledge, this is the largest contemporary study to evaluate sex-based differences in HF hospitalizations and the first large study to characterize sex-based differences in transfer patterns to higher level centers. We found that mortality and transfer to ATCs were significantly lower in females compared to males but were not significantly different after multivariable adjustment for the different comorbidity profiles and HF decompensations. Females were less likely to receive diagnostic and therapeutic interventions across all center types and transfers, even after multivariable adjustment.

## Figures and Tables

**Table 1:** Characteristics of patients admitted with heart failure, stratified by patient sex.

Patient Characteristic	Male (n = 1,473,314)	Female (n = 1,398,953)	*p*-Value * (Absolute Statistical Mean Difference)	Total
**Demographics**				
**Age (years)**	71 (60–81)	76 (65–85)	<0.001 ^†^ (0.299)	74 (62–83)
**Age Group (years)**			<0.001 (0.283)	
<40	44,213 (3.0)	32,735 (2.3)		76,948 (2.7)
40–49	90,948 (6.2)	54,278 (3.9)		145,227 (5.1)
50–59	214,611 (14.6)	133,953 (9.6)		348,564 (12.1)
60–69	321,662 (21.8)	241,559 (17.3)		563,221 (19.6)
70–79	363,580 (24.7)	339,011 (24.2)		702,591 (24.5)
80+	438,301 (29.7)	597,417 (42.7)		1,035,718 (36.1)
**Median Household Income by ZIP Code**			<0.001 (0.011)	
1st Quartile	475,132 (32.7)	459,668 (33.2)		934,800 (33.0)
2nd Quartile	400,770 (27.6)	381,393 (27.6)		782,163 (27.6)
3rd Quartile	337,532 (23.2)	317,634 (23.0)		655,166 (23.1)
4th Quartile	239,543 (16.5)	223,934 (16.2)		463,477 (16.3)
**Comorbidities**				
**Hypertension**	411,778 (27.9)	411,904 (29.4)	<0.001 (0.032)	823,682 (28.7)
**Hyperlipidemia**	801,870 (54.4)	730,493 (52.2)	<0.001 (0.041)	1,532,363 (53.4)
**Coronary Artery Disease**	915,776 (62.2)	673,743 (48.2)	<0.001 (0.284)	1,589,520 (55.3)
**Prior MI**	76,994 (5.2)	64,805 (4.6)	<0.001 (0.152)	141,800 (4.9)
**Atrial Fibrillation/Flutter**	678,527 (46.1)	610,815 (43.7)	<0.001 (0.045)	1,289,342 (44.9)
**Valve Disease**	400,492 (27.2)	437,564 (31.3)	<0.001 (0.092)	838,055 (29.2)
**Peripheral Vascular Disease**	174,273 (11.8)	137,576 (9.8)	<0.001 (0.062)	311,849 (10.9)
**Diabetes**	709,364 (48.1)	643,832 (46.0)	<0.001 (0.043)	1,353,196 (47.1)
**Chronic Kidney Disease**	790,201 (53.6)	659,351 (47.1)	<0.001 (0.130)	1,449,552 (50.5)
**Coagulopathy**	131,998 (9.0)	87,104 (6.2)	<0.001 (0.103)	219,102 (7.6)
**Liver Disease**	109,641 (7.4)	66,925 (4.8)	<0.001 (0.112)	176,566 (6.1)
**Long Term Anticoagulant Use**	339,402 (23.0)	304,288 (21.8)	<0.001 (0.027)	643,690 (22.4)
**Malignancy**	82,384 (5.6)	65,116 (4.7)	<0.001 (0.043)	147,499 (5.1)
**Thyroid Disorder**	186,058 (12.6)	371,085 (26.5)	<0.001 (0.356)	557,142 (19.4)
**Autoimmune Conditions**	29,947 (2.0)	74,268 (5.3)	<0.001 (0.176)	104,215 (3.6)
**Chronic Lung Disease**	535,851 (36.4)	554,056 (39.6)	<0.001 (0.070)	1,089,907 (37.9)
**Malnutrition/Weight Loss**	82,972 (5.6)	88,193 (6.3)	<0.001 (0.028)	171,166 (6.0)
**Tobacco Use**	729,284 (49.5)	473,245 (33.8)	<0.001 (0.321)	1,202,529 (41.9)
**Drug Use**	64,966 (4.4)	28,131 (2.0)	<0.001 (0.139)	93,096 (3.2)
**Alcohol Abuse**	81,175 (5.5)	16,824 (1.2)	<0.001 (0.240)	97,999 (3.4)
**Depression**	128,294 (8.7)	202,514 (14.5)	<0.001 (0.177)	330,808 (11.5)
**Obesity**	366,517 (24.9)	380,938 (27.2)	<0.001 (0.056)	747,455 (26.0)
**Index Presentation**				
**Heart-Failure Decompensations**				
Acute Kidney Injury	478,767 (32.5)	381,743 (27.3)	<0.001 (0.116)	860,511 (30.0)
Mechanical Ventilation/Intubation	34,912 (2.4)	26,092 (1.9)	<0.001 (0.034)	61,004 (2.1)
Non-Invasive Ventilation > 24 h	30,907 (2.1)	32,707 (2.3)	<0.001 (0.019)	63,614 (2.2)
Cardiogenic Shock	39,792 (2.7)	20,817 (1.5)	<0.001 (0.085)	60,610 (2.1)
Cardiac Arrest	19,392 (1.3)	12,753 (0.9)	<0.001 (0.039)	32,144 (1.1)
Ventricular Arrhythmias	106,285 (7.2)	47,597 (3.4)	<0.001 (0.171)	153,883 (5.4)
**Myocardial Infarction**	76,994 (5.2)	64,805 (4.6)	<0.001 (0.027)	141,800 (4.9)
STEMI	5060 (0.3)	4124 (0.3)	<0.001 (0.010)	9184 (0.3)
**Stroke/TIA**	10,535 (0.7)	10,621 (0.8)	0.003 (0.006)	21,157 (0.7)
**Weekend Admission**	335,608 (22.8)	331,741 (23.7)	<0.001 (0.022)	667,349 (23.2)
**Elective Admission**	71,783 (4.9)	59,281 (4.2)	<0.001 (0.033)	131,064 (4.6)
**Primary Payer**			<0.001 (0.241)	
Medicare	1,037,329 (70.5)	1,121,798 (80.3)		2,159,128 (75.3)
Medicaid	154,652 (10.5)	113,832 (8.1)		268,485 (9.4)
Private Insurance	188,815 (12.8)	123,755 (8.9)		312,570 (10.9)
Self-Pay/No Charge/Other	90,681 (6.2)	38,245 (2.7)		128.926 (4.5)
**Disposition Status**			<0.001	
Home/Home Health Care	1,183,531 (80.3)	1,042,650 (74.5)		2,226,180 (77.5)
Transfer to SNF/ICF/Other	221,001 (15.0)	309,011 (22.1)		530,011 (18.5)
AMA/Unknown/Other	28,254 (1.9)	11,002 (0.8)		39,255 (1.4)
Died	40,529 (2.8)	36,292 (2.6)		76,821 (2.7)
**Transfer to ATC**	9389 (0.6)	6152 (0.4)	<0.001	15,541 (0.5)
	(60.4% of all transfers)	(39.6% of all transfers)		

Values are presented as numbers (percentage) for categorical values and medians (interquartile range) for continuous variables. * The Rao–Scott χ^2^ test was used for all statistical tests in comparison to male patients unless stated otherwise. ^†^ The Mann–Whitney–Wilcoxon nonparametric test was used. **Following abbreviations apply:** MI (Myocardial Infarction); STEMI (ST-elevation Myocardial Infarction); TIA (Transient Ischemic Attack); SNF (Skilled Nursing Facility); ICF (Intermediate Care Facility); ATC (Advanced Therapy Center).

**Table 2:** Characteristics of patients admitted with heart failure, stratified by patient sex, hospital type, and transfer.

Patient Characteristic	Direct to Non-ATC (Group A) n = 2,331,690			Direct to ATC (Group B) n = 525,037			Transfer to ATC (Group C) n = 15,541		
	Male	Female	*p*-Value *	Male	Female	*p*-Value *	Male	Female	*p*-Value *
Demographics	n = 1,184,082 (50.8)	n = 1,147,608 (49.2)		n = 279,844 (53.3)	n = 245,193 (46.7)		n = 9389 (60.4)	n = 6152 (39.6)	
**Age (years)**	72 (60–81)	77 (66–86)	<0.001 ^†^	68 (57–78)	73 (62–84)	<0.001 ^†^	63 (53–73)	66 (55–75)	<0.001 ^†^
**Age Group (years)**			<0.001			<0.001			<0.001
<40	31,205 (2.6)	23,032 (2.0)		12,247 (4.4)	9119 (3.7)		761 (8.1)	584 (9.5)	
40–49	68,548 (5.8)	41,345 (3.6)		21,423 (7.7)	12,448 (5.1)		977 (10.4)	485 (7.9)	
50–59	164,852 (13.9)	104,302 (9.1)		47,884 (17.1)	28,710 (11.7)		1874 (20.0)	942 (15.3)	
60–69	252,298 (21.3)	191,974 (16.7)		66,721 (23.8)	48,031 (19.6)		2643 (28.2)	1555 (25.3)	
70–79	296,840 (25.1)	279,606 (24.4)		64,757 (23.1)	57,872 (23.6)		1983 (21.1)	1533 (24.9)	
80+	370,339 (31.3)	507,349 (44.2)		66,812 (23.9)	89,014 (36.3)		1150 (12.3)	1053 (17.1)	
**Median Household Income by ZIP Code**			0.128			<0.001			0.039
1st Quartile	383,273 (32.8)	374,878 (33.1)		89,573 (32.4)	83,121 (34.2)		2286 (24.7)	1668 (27.5)	
2nd Quartile	330,722 (28.3)	320,618 (28.3)		67,363 (24.3)	59,032 (24.3)		2685 (29.0)	1742 (28.8)	
3rd Quartile	268,413 (23.0)	258,831 (22.8)		66,729 (24.1)	57,288 (23.6)		2390 (25.8)	1515 (25.0)	
4th Quartile	184,641 (15.8)	179,185 (15.8)		52,993 (19.2)	43,618 (17.9)		1909 (20.6)	1131 (18.7)	
**Comorbidities**									
**Hypertension**	329,675 (27.8)	337,152 (29.4)	<0.001	77,349 (27.6)	71,554 (29.2)	<0.001	4754 (50.6)	3199 (52.0)	0.291
**Hyperlipidemia**	647,074 (54.6)	600,035 (52.3)	<0.001	149,356 (53.4)	127,019 (51.8)	<0.001	5440 (57.9)	3439 (55.9)	0.082
**Coronary Artery Disease**	734,940 (62.1)	551,340 (48.0)	<0.001	173,889 (62.1)	118,453 (48.3)	<0.001	6948 (74.0)	3950 (64.2)	<0.001
**Prior MI**	58,841 (5.0)	50,699 (4.4)	<0.001	16,290 (5.8)	12,727 (5.2)	<0.001	1863 (19.8)	1379 (22.4)	0.020
**Atrial Fibrillation/Flutter**	545,791 (46.1)	504,815 (44.0)	<0.001	127,900 (45.7)	103,420 (42.2)	<0.001	4835 (51.5)	2579 (41.9)	<0.001
**Coagulopathy**	100,055 (8.5)	67,651 (5.9)	<0.001	29,630 (10.6)	18,353 (7.5)	<0.001	2313 (24.6)	1100 (17.9)	<0.001
**Valve Disease**	316,636 (26.7)	354,246 (30.9)	<0.001	79,333 (28.3)	80,191 (32.7)	<0.001	4523 (48.2)	3127 (50.8)	0.036
**Peripheral Vascular Disease**	140,955 (11.9)	113,464 (9.9)	<0.001	31,744 (11.3)	23,279 (9.5)	<0.001	1573 (16.8)	832 (13.5)	<0.001
**Diabetes**	573,344 (48.4)	527,858 (46.0)	<0.001	131,781 (47.1)	113,222 (46.2)	0.001	4239 (45.2)	2752 (44.7)	0.702
**Chronic Kidney Disease**	626,532 (52.9)	535,934 (46.7)	<0.001	158,347 (56.6)	120,574 (49.2)	<0.001	5322 (56.7)	2843 (46.2)	<0.001
**Liver Disease**	82,168 (6.9)	50,723 (4.4)	<0.001	25,583 (9.1)	15,348 (6.3)	<0.001	1890 (20.1)	854 (13.9)	<0.001
**Long Term Anticoagulant Use**	271,301 (22.9)	249,012 (21.7)	<0.001	65,546 (23.4)	53,849 (22.0)	<0.001	2555 (27.2)	1427 (23.2)	<0.001
**Malignancy**	65,116 (5.5)	51,906 (4.5)	<0.001	16,727 (6.0)	12,816 (5.2)	<0.001	540 (5.8)	394 (6.4)	0.210
**Thyroid Disorder**	150,471 (12.7)	307,288 (26.8)	<0.001	34,232 (12.2)	62,004 (25.3)	<0.001	1355 (14.4)	1793 (29.1)	<0.001
**Autoimmune Conditions**	23,883 (2.0)	59,168 (5.2)	<0.001	5787 (2.1)	14,597 (6.0)	<0.001	276 (2.9)	503 (8.2)	<0.001
**Chronic Lung Disease**	446,346 (37.7)	462,663 (40.3)	<0.001	86,285 (30.8)	88,872 (36.2)	<0.001	3220 (34.3)	2521 (41.0)	<0.001
**Malnutrition/Weight Loss**	61,667 (5.2)	69,076 (6.0)	<0.001	19,848 (7.1)	18,180 (7.4)	0.011	1457 (15.5)	937 (15.2)	0.736
**Tobacco Use**	593,154 (50.1)	389,625 (34.0)	<0.001	130,761 (46.7)	80,927 (33.0)	<0.001	5369 (57.2)	2693 (43.8)	<0.001
**Drug Use**	51,587 (4.4)	22,559 (2.0)	<0.001	12,839 (4.6)	5322 (2.2)	<0.001	539 (5.7)	250 (4.1)	0.002
**Alcohol Abuse**	65,152 (5.5)	13,732 (1.2)	<0.001	15,016 (5.4)	2927 (1.2)	<0.001	1007 (10.7)	165 (2.7)	<0.001
**Depression**	101,506 (8.6)	164,840 (14.4)	<0.001	25,570 (9.1)	36,356 (14.8)	<0.001	1219 (13.0)	1318 (21.4)	<0.001
**Obesity**	295,252 (24.9)	309,965 (27.0)	<0.001	68,545 (24.5)	69,001 (28.1)	<0.001	2721 (29.0)	1972 (32.0)	0.003
**Index Presentation**									
**Heart-Failure Decompensations**									
Acute Kidney Injury	367,156 (31.0)	300,342 (26.2)	<0.001	105,649 (37.8)	78,226 (31.9)	<0.001	5962 (63.5)	3175 (51.6)	<0.001
Mechanical Ventilation/Intubation	23,625 (2.0)	18,483 (1.6)	<0.001	9590 (3.4)	6551 (2.7)	<0.001	1698 (18.1)	1057 (17.2)	0.373
Non-Invasive Ventilation > 24 h	25,054 (2.1)	27,043 (2.4)	<0.001	5517 (2.0)	5429 (2.2)	<0.001	335 (3.6)	235 (3.8)	0.611
Cardiogenic Shock	19,081 (1.6)	11,247 (1.0)	<0.001	17,179 (6.1)	7938 (3.2)	<0.001	3532 (37.6)	1632 (26.5)	<0.001
Cardiac Arrest	13,893 (1.2)	9418 (0.8)	<0.001	4678 (1.7)	2894 (1.2)	<0.001	821 (8.7)	441 (7.2)	0.013
Ventricular Arrhythmias	73,875 (6.2)	34,140 (3.0)	<0.001	29,466 (10.5)	12,408 (5.1)	<0.001	2944 (31.4)	1049 (17.0)	<0.001
**Myocardial Infarction**	58,841 (5.0)	50,699 (4.4)	<0.001	16,290 (5.8)	12,727 (5.2)	<0.001	1863 (19.8)	1379 (22.4)	0.020
STEMI	3599 (0.3)	3072 (0.3)	<0.001	1106 (0.4)	804 (0.3)	0.005	355 (3.8)	247 (4.0)	0.581
**Stroke/TIA**	7652 (0.6)	8240 (0.7)	<0.001	2569 (0.9)	2234 (0.9)	0.865	314 (3.3)	148 (2.4)	0.024
**Weekend Admission**	274,140 (23.2)	275,313 (24.0)	<0.001	59,341 (21.2)	55,069 (22.5)	<0.001	2127 (22.7)	1358 (22.1)	0.538
**Elective Admission**	52,548 (4.4)	47,191 (4.1)	<0.001	18,596 (6.7)	11,678 (4.8)	<0.001	639 (6.8)	412 (6.7)	0.880
**Primary Payer**			<0.001			<0.001			<0.001
Medicare	846,750 (71.6)	930,679 (81.2)		185,326 (66.3)	187,160 (76.4)		5253 (56.0)	3959 (64.4)	
Medicaid	121,125 (10.2)	89,280 (7.8)		32,232 (11.5)	23,788 (9.7)		1295 (13.8)	764 (12.4)	
Private Insurance	141,519 (12.0)	95,612 (8.3)		44,944 (16.1)	26,945 (11.0)		2352 (25.1)	1198 (19.5)	
Self-Pay/No Charge/Other	73,306 (6.2)	30,977 (2.7)		16,886 (6.0)	7037 (2.9)		489 (5.2)	231 (3.8)	
**Disposition Status**			<0.001			<0.001			<0.001
Home/Home Health Care	945,365 (79.8)	848,263 (73.9)		231,254 (82.6)	190,095 (77.5)		6912 (73.6)	4292 (69.8)	
Transfer to SNF/ICF/Other	184,131 (15.6)	261,019 (22.7)		35,581 (12.7)	46,786 (19.1)		1288 (13.7)	1205 (19.6)	
AMA/Unknown/Other	23,644 (2.0)	9387 (0.8)		4531 (1.6)	1584 (0.6)		79 (0.8)	30 (0.5)	
Died	30,942 (2.6)	28,940 (2.5)		8478 (3.0)	6727 (2.7)		1109 (11.8)	625 (10.2)	

Values are presented as numbers (percentage) for categorical values and weighted medians (interquartile range) for continuous variables. * The Rao–Scott χ^2^ test was used for all statistical tests in comparison to male patients unless stated otherwise. ^†^ The Mann–Whitney–Wilcoxon nonparametric test was used. **Following abbreviations apply:** MI (Myocardial Infarction); STEMI (ST-elevation Myocardial Infarction); TIA (Transient Ischemic Attack); SNF (Skilled Nursing Facility); ICF (Intermediate Care Facility); ATC (Advanced Therapy Center).

**Table 3:** Outcomes and procedures performed for index heart-failure admissions, stratified by patient sex, hospital type, and transfer.

Patient Characteristic	Direct to Non-ATC (Group A) n = 2,331,690			Direct to ATC (Group B) n = 525,037			Transfer to ATC (Group C) n = 15,541		
	Male	Female	*p*-Value	Male	Female	*p*-Value	Male	Female	*p*-Value
	1,184,082 (50.8)	1,147,608 (49.2)		279,844 (53.3)	245,193 (46.7)		9389 (60.4)	6152 (39.6)	
Index Mortality	30,942 (2.6)	28,940 (2.5)	0.002	8478 (3.0)	6727 (2.7)	<0.001	1109 (11.8)	625 (10.2)	0.044
Length of Stay, days	3.2 (1.8– 5.4)	3.3 (2.0– 5.5)	<0.001	4.0 (2.2– 7.2)	4.0 (2.3– 6.9)	<0.001	11.0 (6.5–18.6)	9.4 (5.7– 15.8)	<0.001
Estimated Cost, $USD	7773 (4975–12,766)	7758 (5107–12,321)	0.002	9739 (5763–18,313)	9128 (5680–15,851)	<0.001	32,154 (17,403–64,587)	25,418 (14,832–48,321)	<0.001
90-Day Readmission among Survivors	425,869 (36.9)	409,234 (36.6)	<0.001	103,755 (38.2)	90,412 (37.9)	0.152	3167 (38.3)	2030 (36.7)	0.233
**Procedures Performed**									
CRT/ICD placement	20,261 (1.7)	8972 (0.8)	<0.001	9062 (3.2)	4239 (1.7)	<0.001	839 (8.9)	352 (5.7)	<0.001
PCI	16,230 (1.4)	9800 (0.9)	<0.001	4396 (1.8)	2884 (1.2)	<0.001	752 (8.0)	353 (5.7)	<0.001
RHC	42,538 (3.6)	30,868 (2.7)	<0.001	39,367 (14.1)	22,346 (9.1)	<0.001	4608 (49.1)	2342 (38.1)	<0.001
CABG	2669 (0.2)	1194 (0.1)	<0.001	1367 (0.5)	607 (0.2)	<0.001	242 (2.6)	96 (1.6)	0.003
TAVR	413 (<0.1)	293 (<0.1)	0.005	438 (0.2)	314 (0.1)	0.057	43 (0.5)	25 (0.4)	0.724
Temporary MCS	2913 (0.2)	1471 (0.1)	<0.001	5891 (2.1)	2043 (0.8)	<0.001	1546 (16.5)	644 (10.5)	<0.001
IABP	1569 (0.1)	858 (0.1)	<0.001	3077 (1.1)	1068 (0.4)	<0.001	887 (9.4)	382 (6.2)	<0.001
PVAD	1390 (0.1)	601 (0.1)	<0.001	1333 (0.5)	447 (0.2)	<0.001	540 (5.7)	217 (3.5)	<0.001
ECMO	55 (<0.1)	32 (<0.1)	0.120	1034 (0.4)	432 (0.2)	<0.001	390 (4.2)	189 (3.1)	0.042
LVAD *	Not Performed	Not Performed	NA	3491 (1.2)	994 (0.4)	<0.001	454 (4.8)	95 (1.5)	<0.001
Heart Transplant *	Not Performed	Not Performed	NA	3592 (1.3)	1193 (0.5)	<0.001	86 (0.9)	30 (0.5)	0.058

Values are presented as numbers (percentages) for categorical variables and medians (interquartile range) for continuous variables. * LVAD and heart transplants were not performed in non-ATCs. **Following abbreviations apply:** ATC (Advanced Therapy Center); CRT/ICD placement (Cardiac Resynchronization Therapy/Implantable Cardioverter-Defibrillator placement); PCI (Percutaneous Coronary Intervention); RHC (Right Heart Catheterization); CABG (Coronary Artery Bypass Grafting); TAVR (Transcatheter Aortic Valve Replacement); Temporary MCS (Mechanical Circulatory Support); IABP (Intra-Aortic Balloon Pump); PVAD (Percutaneous Ventricular Assist Device); ECMO (Extracorporeal Membrane Oxygenation); LVAD (Left Ventricular Assist Device).

**Table 4:** Univariable and multivariable analysis of the association of female sex with heart-failure management and outcomes.

Characteristic	Odds Ratio (Univariable)	*p* Value (Univariable)	Odds Ratio (Multivariable)	*p* Value (Multivariable)
Index Mortality	0.94 (0.92–0.96)	<0.001	0.99 (0.96–1.02)	0.404
90-day Readmission	0.99 (0.98–0.99)	<0.001	1.03 (1.02–1.04)	<0.001
**Procedures Performed**				
RHC	0.66 (0.65–0.68)	<0.001	0.87 (0.85–0.89)	<0.001
Any MCS	0.42 (0.40–0.45)	<0.001	0.72 (0.68–0.77)	<0.001
CABG	0.47 (0.43–0.51)	<0.001	0.76 (0.70–0.83)	<0.001
PCI	0.62 (0.60–0.64)	<0.001	0.84 (0.81–0.87)	<0.001
TAVR	0.74 (0.64–0.86)	<0.001	0.76 (0.66–0.89)	<0.001
IABP	0.44 (0.40–0.48)	<0.001	0.80 (0.73–0.87)	<0.001
PVAD	0.41 (0.37–0.45)	<0.001	0.69 (0.62–0.77)	<0.001
ECMO	0.47 (0.41–0.53)	<0.001	0.89 (0.76–1.04)	0.144
CRT/ICD	0.47 (0.46–0.48)	<0.001	0.61 (0.59–0.63)	<0.001
LVAD *	0.29 (0.26–0.33)	<0.001	0.58 (0.51–0.67)	<0.001
Transplant *	0.35 (0.32–0.38)	<0.001	0.63 (0.56–0.70)	<0.001

* LVAD transplants were not performed in non-ATCs. **Following abbreviations apply:** CRT/ICD placement (Cardiac Resynchronization Therapy/Implantable Cardioverter-Defibrillator placement); PCI (Percutaneous Coronary Intervention); RHC (Right Heart Catheterization); CABG (Coronary Artery Bypass Grafting); TAVR (Transcatheter Aortic Valve Replacement); Temporary MCS (Mechanical Circulatory Support); IABP (Intra-Aortic Balloon Pump); PVAD (Percutaneous Ventricular Assist Device); ECMO (Extracorporeal Membrane Oxygenation); LVAD (Left Ventricular Assist Device); ATC (Advanced Therapy Center).

**Table 5:** Etiology and classification of heart failure by ICD10 codes among index admissions, stratified by patient sex.

Heart-Failure Etiology/Classification	Total	Male	Female	*p* Value
Ischemic Cardiomyopathy	735,922 (25.6)	460,180 (31.2)	275,742 (19.7)	<0.001
Dilated Cardiomyopathy	161,552 (5.6)	106,671 (7.2)	54,881 (3.9)	<0.001
Hypertrophic Cardiomyopathy	25,601 (0.9)	10,764 (0.7)	14,837 (1.1)	<0.001
Restrictive Cardiomyopathy	4592 (0.2)	2421 (0.2)	2171 (0.2)	0.199
Hypertensive Heart Failure	2,031,430 (70.7)	1,049,135 (71.2)	982,295 (70.2)	<0.001
Diastolic Heart Failure	1,153,864 (40.2)	458,022 (31.1)	695,842 (49.7)	<0.001
Right Heart Failure	23,361 (0.8)	11,312 (0.8)	12,049 (0.9)	<0.001
Myocarditis	6058 (0.2)	3393 (0.2)	2665 (0.2)	<0.001
Takotsubo Cardiomyopathy	27,692 (1.0)	2920 (0.2)	24,771 (1.8)	<0.001
Peripartum Cardiomyopathy	5593 (0.2)	0 (0.0)	5593 (0.4)	N/A
Sarcoid Cardiomyopathy	1028 (<0.1)	567 (<0.1)	461 (<0.1)	0.061
Rheumatic Heart Failure	1560 (<0.1)	479 (<0.1)	1081 (<0.1)	<0.001
Alcohol Cardiomyopathy	13,880 (0.5)	12,253 (0.8)	1627 (0.1)	<0.001
Drug-Induced Cardiomyopathy	11,921 (0.4)	7047 (0.5)	4874 (0.3)	<0.001
High Output Heart Failure	620 (<0.1)	257 (<0.1)	362 (<0.1)	<0.001

See Table S1 for ICD codes used for each etiology/classification.

## Data Availability

The data used in this study are from the Healthcare Cost and Utilization Project (HCUP) Nationwide Readmissions Database (NRD). These data are publicly available but require completion of a data use agreement and purchase through HCUP (https://www.hcup-us.ahrq.gov/). The authors do not have permission to share the data.

## Supplementary Materials

The following supporting information can be downloaded at https://www.mdpi.com/article/10.3390/jcm15072776/s1, Supplementary Table S1: ICD-10-CM and procedure codes used to define heart failure, comorbidities, etiologies, clinical events, and procedures. Supplementary Table S2A: Multivariable analysis for predictors of index mortality among admissions for heart failure. Supplementary Table S2B: Sensitivity analysis for predictors of mortality among index admission for heart failure, with additional adjustment for HF-related procedures. Supplementary Table S2C: Multivariable analysis for predictors of transfer to an ATC among index admission for heart failure. Supplementary Table S3: Multivariable analysis for predictors of utilization of right heart catheterization and temporary mechanical circulatory support among index admissions for heart failure. Supplementary Table S4: Sensitivity analysis of outcomes and procedures performed for index heart-failure admissions, stratified by hospital type based on high-volume ATCs, transfer status, and patient sex. Supplementary Table S5: Sensitivity analysis of the association of female sex with heart-failure management and outcomes in context of high-volume advanced therapy centers. Supplementary Table S6: Characteristics of patients with severe heart failure stratified by sex, hospital type, and transfer status. Supplementary Table S7: Outcomes and procedures performed for index admission for severe heart failure, stratified by patient sex, hospital type, and transfer. Supplementary Table S8: Multivariable analysis of the association of female sex with heart failure management and outcomes among patients admitted for severe heart failure. Supplementary Table S9: Subgroup analyses of the association of sex with procedural utilization, with multivariable models adjusted for demographic, clinical, and hospital characteristics as described in the primary analysis. Supplementary Figure S1: Frequencies of procedure utilization, stratified by patient sex, hospitalization type, and transfer.
