# Supplementary material for: Sex-Based Differences in Management and Outcomes of Patients Admitted or Transferred to Advanced Therapy Centers for Heart Failure
Source: J Clin Med. 2026 Apr 7;15(7):2776. doi: 10.3390/jcm15072776 (PMC13073529; doi:10.3390/jcm15072776)
Supplement: Supplementary file 1 [file jcm-15-02776-s001.zip › jcm-4197734-supplementary.pdf]

SUPPLEMENTAL MATERIAL

Table S1. ICD10-CM codes

Condition	ICD Codes
Heart Failure and Cardiomyopathy	I110, I130, I132, I0981, I501, I5020, I5021, I5022, I5023, I5030, I5031, I5032, I5033, I5040, I5041, I5042, I5043, I50810, I50811, I50812, I50813, I50814, I5082, I5083, I5084, I5089, I509, I5181, I97130, I97131, O29121, O29122, O29123, O29129, R570, T8111XA, A3681, B3324, D8685, I255, I420, I421, I422, I423, I424, I425, I426, I427, I428, I429, I43, O903
Hyperlipidemia	E780, E7800, E7801, E781, E782, E783, E784, E7841, E7849, E785
Coronary Artery Disease	I200, I201, I208, I209, I240, I248, I249, I2510, I25110, I25111, I25118, I25119, I252, I255, I256, I25700, I25701, I25708, I25709, I25710, I25711, I25718, I25719, I25720, I25721, I25728, I25729, I25730, I25731, I25738, I25739, I25750, I25751, I25758, I25759, I25760, I25761, I25768, I25769, I25790, I25791, I25798, I25799, I25810, I25811, I25812, I2582, I2583, I2584, I2589, I259, Z951, Z955, Z9861, T82211A, T82211D, T82211S, T82212A, T82212D, T82212S, T82213A, T82213D, T82213S, T82218A, T82218D, T82218S, T82855A, T82855D, T82855S, I2101, I2102, I2109, I2111, I2119, I2121, I2129, I213, I220, I221, I228, I229, I214, I219, I21A1, I21A9, I222
Prior MI	I252
Atrial Fibrillation/Aflutter	I480, I481, I4811, I4819, I482, I4820, I4821, I483, I484, I4891, I4892
Valve Disease/Endocarditis	A1884, A3282, A3951, A5203, B3321, B376, I011, I018, I019, I020, I050, I051, I052, I058, I059, I060, I061, I062, I068, I069, I070, I071, I072, I078, I079, I080, I081, I082, I083, I088, I089, I091, I0989, I330, I339, I340, I341, I342, I348, I349, I350, I351, I352, I358, I359, I360, I361, I362, I368, I369, I370, I371, I372, I378, I379, I38, I39, M3211, Q220, Q221, Q222, Q223, Q224, Q225, Q226, Q228, Q229, Q230, Q231, Q232, Q233, Q234, Q238, Q239, T8201XA, T8201XD, T8201XS, T8202XA, T8202XD, T8202XS, T8203XA, T8203XD, T8203XS, T8209XA, T8209XD, T8209XS, T82221A, T82221D, T82221S, T82222A, T82222D, T82222S, T82223A, T82223D, T82223S, T82228A, T82228D, T82228S, T826XXA, T826XXD, T826XXS, Z952, Z953, Z954
Chronic Kidney Disease	N181, N182, N183, N1830, N1831, N1832, N184, N185, N186, N189, D631, E0822, E0922, E1022, E1122, E1322, I120, I129, I130, I1310, I1311, I132, O10211, O10212, O10213, O10219, O1022, O1023, O10311, O10312, O10313, O10319, O1032, O1033, T8612, Z9115, Z992
Liver Disease	B190, B1911, B1921, I8500, I8501 I8510, I8511, I864, K7040, K7041 K7210, K7211, K7290, K7291, K765 K766, K767, K9182, A5145, A5274 B180, B181, B182, B188, B189 B1910, B1920, B199, B251, B581 K700, K7010, K7011, K702, K7030 K7031, K709, K713, K714, K7150 K7151, K716, K717, K718, K730 K731, K732, K738, K739, K740 K7400, K7401, K7402, K741, K742 K743, K744, K745, K7460, K7469 K751, K752, K753, K754, K7581 K7589, K759, K760, K761, K762 K763, K764, K7681, K7689, K769 K77, T8642
Coagulopathy	D6109, D611, D612, D613, D61810, D61811, D61818, D6182, D6189, D619, D65, D66, D67, D680, D681, D682, D68311, D68312, D68318, D6832, D684, D688, D689, D691, D693, D6941, D6942, D6949, D6951, D6959, D696, D698, D699, D7582, O99111, O99112, O99113, O99119, O9912, O9913
Long Term Anticoagulant Use	Z7901
Malnutrition	E40, E41, E42, E43, E440, E441, E45, E46, E640, O2510, O2511, O2512, O2513, O252, O253, R634, R636, R64
Smoking/Tobacco Use	F17200, F17201, F17203, F17208, F17209, F17210, F17211, F17213, F17218, F17219, F17220, F17221, F17223, F17228, F17229, F17290, F17291, F17293, F17298, F17299, O99330, O99331, O99332, O99333, O99334, O99335, Z720, Z87891, Z5301, T65211A, T65211D, T65211S, T65212A, T65212D, T65212S, T65213A, T65213D, T65213S, T65214A, T65214D, T65214S, T65221A, T65221D, T65221S, T65222A, T65222D, T65222S, T65223A, T65223D, T65223S, T65224A, T65224D, T65224S, T65291A, T65291D, T65291S, T65292A, T65292D, T65292S, T65293A, T65293D, T65293S, T65294A, T65294D, T65294S
**Etiologies of Heart Failure**	
Hypertensive Heart Failure	I110, I130, I132
Diastolic Heart Failure	I5030, I5031, I5032, I5033
Right Heart Failure	I50810, I50811, I50812, I50813, I50814
Dilated Cardiomyopathy	I420
Hypertrophic Cardiomyopathy	I421, I422
Restrictive Cardiomyopathy	I425
Ischemic Cardiomyopathy	I214, I219, I21A1, I21A9, I222, I2101, I2102, I2109, I2111, I2119, I2121, I2129, I213, I220, I221, I228, I229, I255, I252
Rheumatic Heart Failure	I0981
High Output Heart Failure	I5083
Alcohol Cardiomyopathy	I426
Drug-Induced Cardiomyopathy	I427
Myocarditis	A3681, B3324, D8685, A381, A3952, B2682, B3322, B570, B5881, I012, I090, I400, I401, I408, I409, I41, I514, J1082, J1182
Takotsubo	I5181
Sarcoid Cardiomyopathy	D8685
Peripartum Cardiomyopathy	O903
**Events**	

Stroke/TIA	G9511, G450, G451, G452, G453, G454, G458, G459, G460, G461<br>G462, G463, G464, G465, G466, G467, G468, H3400, H3401, H3402<br>H3403, H3410, H3411, H3412, H3413, H34211, H34212, H34213, H34219, H34231<br>H34232, H34233, H34239, H93011, H93012, H93013, H93019, I6000, I6001, I6002<br>I6010, I6011, I6012, I602, I6020, I6021, I6022, I6030, I6031, I6032<br>I604, I6050, I6051, I6052, I606, I607, I608, I609, I610, I611<br>I612, I613, I614, I615, I616, I618, I619, I6200, I6201, I6202<br>I621, I629, I6300, I63011, I63012, I63013, I63019, I6302, I63031, I63032<br>I63033, I63039, I6309, I6310, I63111, I63112, I63113, I63119, I6312, I63131<br>I63132, I63133, I63139, I6319, I6320, I63211, I63212, I63213, I63219, I6322<br>I63231, I63232, I63233, I63239, I6329, I6330, I63311, I63312, I63313, I63319<br>I63321, I63322, I63323, I63329, I63331, I63332, I63333, I63339, I63341, I63342<br>I63343, I63349, I6339, I6340, I63411, I63412, I63413, I63419, I63421, I63422<br>I63423, I63429, I63431, I63432, I63433, I63439, I63441, I63442, I63443, I63449<br>I6349, I6350, I63511, I63512, I63513, I63519, I63521, I63522, I63523, I63529<br>I63531, I63532, I63533, I63539, I63541, I63542, I63543, I63549, I6359, I636<br>I638, I6381, I6389, I639, I6781, I6782, I97810, I97811, I97820, I97821<br>G43601, G43609, G43611, G43619, R29701, R29702, R29703, R29704, R29705, R29706<br>R29707, R29708, R29709, R29710, R29711, R29712, R29713, R29714, R29715, R29716<br>R29717, R29718, R29719, R29720, R29721, R29722, R29723, R29724, R29725, R29726<br>R29727, R29728, R29729, R29730, R29731, R29732, R29733, R29734, R29735, R29736<br>R29737, R29738, R29739, R29740, R29741, R29742,<br>0W300ZZ, 0W303ZZ, 0W304ZZ, 0W310ZZ, 0W313ZZ, 0W314ZZ
Acute Kidney Injury	N170, N171, N172, N178, N179, N990
Ventricular Arrhythmia	I470, I472, I4901, I4902
Non-invasive Ventilation Support ≥24 hours	5A09457, 5A09458, 5A09459, 5A0945A, 5A0945B, 5A0945Z, 5A09557, 5A09558, 5A09559, 5A0955A, 5A0955B, 5A0955Z, 5A19054
STEMI	I2101, I2102, I2109, I2111, I2119, I2121, I2129, I213, I220, I221, I228, I229
Cardiogenic Shock	R570, T8111XA
Intubation/Mechanical Ventilation	5A1935Z, 5A1945Z, 5A1955Z, 0BH17EZ, 0BH18EZ, 09HN7BZ, 09HN8BZ, 0CHY7BZ, 0CHY8BZ
Cardiac Arrest / CPR	I462, I468, I469, I97120, I97121, I97710, I97711, 5A12012, 5A1221J, I4901
**Procedures Performed**	
ICD/CRT Placement	0JH607Z, 0JH609Z, 0JH637Z, 0JH639Z, 0JH807Z, 0JH809Z, 0JH837Z, 0JH839Z, 0JH638Z, 0JH808Z, 0JH838Z, 0JH608Z, 0JH60FZ, 0JH63FZ
PCI	0210344, 02103D4, 0211344, 02113D4, 0212344, 02123D4, 0213344, 02133D4, 0270346, 027034Z, 0270356, 027035Z, 0270366, 027036Z, 0270376, 027037Z, 02703D6, 02703DZ, 02703E6, 02703EZ, 02703F6, 02703FZ, 02703G6, 02703GZ, 02703T6, 02703TZ, 02703Z6, 02703ZZ, 0271346, 027134Z, 0271356, 027135Z, 0271366, 027136Z, 0271376, 027137Z, 02713D6, 02713DZ, 02713E6, 02713EZ, 02713F6, 02713FZ, 02713G6, 02713GZ, 02713T6, 02713TZ, 02713Z6, 02713ZZ, 0272346, 027234Z, 0272356, 027235Z, 0272366, 027236Z, 0272376, 027237Z, 02723D6, 02723DZ, 02723E6, 02723EZ, 02723F6, 02723FZ, 02723G6, 02723GZ, 02723T6, 02723TZ, 02723Z6, 02723ZZ, 0273346, 027334Z, 0273356, 027335Z, 0273366, 027336Z, 0273376, 027337Z, 02733D6, 02733DZ, 02733E6, 02733EZ, 02733F6, 02733FZ, 02733G6, 02733GZ, 02733T6, 02733TZ, 02733Z6, 02733ZZ, 02C03Z6, 02C03Z7, 02C03ZZ, 02C13Z6, 02C13Z7, 02C13ZZ, 02C23Z6, 02C23Z7, 02C23ZZ, 02C33Z6, 02C33Z7, 02C33ZZ, 02F03ZZ, 02F13ZZ, 02F23ZZ, 02F33ZZ, 02H03DZ, 02H03YZ, 02H13DZ, 02H13YZ, 02H23DZ, 02H23YZ, 02H33DZ, 02H33YZ, 02N03ZZ, 02N13ZZ, 02N23ZZ, 02N33ZZ, 02Q03ZZ, 02Q13ZZ, 02Q23ZZ, 02Q33ZZ, 02U037Z, 02U038Z, 02U03JZ, 02U03KZ, 02U137Z, 02U138Z, 02U13JZ, 02U13KZ, 02U237Z, 02U238Z, 02U23JZ, 02U23KZ, 02U337Z, 02U338Z, 02U33JZ, 02U33KZ, X2C0361, X2C1361, X2C2361, X2C3361
Right Heart Catheterization, Swan Ganz Catheter	02HP30Z, 02HP32Z, 02HQ30Z, 02HQ32Z, 02HR30Z, 02HR32Z, 4A0239Z, 4A023N6, 4A023N8, 4A03353, 4A033B3, 4A1239Z, 4A13353, 4A133B3
TAVR	02RF37H, 02RF37Z, 02RF38H, 02RF38Z, 02RF3JH, 02RF3JZ, 02RF3KH, 02RF3KZ, X2RF332
CABG	0210083, 0210088, 0210089, 021008C, 021008F, 021008W, 0210093, 0210098, 0210099, 021009C, 021009F, 021009W, 02100A3, 02100A8, 02100A9, 02100AC, 02100AF, 02100AW, 02100J3, 02100J8, 02100J9, 02100JC, 02100JF, 02100JW, 02100K3, 02100K8, 02100K9, 02100KC, 02100KF, 02100KW, 02100Z3, 02100Z8, 02100Z9, 02100ZC, 02100ZF, 0211083, 0211088, 0211089, 021108C, 021108F, 021108W, 0211093, 0211098, 0211099, 021109C, 021109F, 021109W, 02110A3, 02110A8, 02110A9, 02110AC, 02110AF, 02110AW, 02110J3, 02110J8, 02110J9, 02110JC, 02110JF, 02110JW, 02110K3, 02110K8, 02110K9, 02110KC, 02110KF, 02110KW, 02110Z3, 02110Z8, 02110Z9, 02110ZC, 02110ZF, 0212083, 0212088, 0212089, 021208C, 021208F, 021208W, 0212093, 0212098, 0212099, 021209C, 021209F, 021209W, 02120A3, 02120A8, 02120A9, 02120AC, 02120AF, 02120AW, 02120J3, 02120J8, 02120J9, 02120JC, 02120JF, 02120JW, 02120K3, 02120K8, 02120K9, 02120KC, 02120KF, 02120KW, 02120Z3, 02120Z8, 02120Z9, 02120ZC, 02120ZF, 0213083, 0213088, 0213089, 021308C, 021308F, 021308W, 0213093, 0213098, 0213099, 021309C, 021309F, 021309W, 02130A3, 02130A8, 02130A9, 02130AC, 02130AF, 02130AW, 02130J3, 02130J8, 02130J9, 02130JC, 02130JF, 02130JW, 02130K3, 02130K8, 02130K9, 02130KC, 02130KF, 02130KW, 02130Z3, 02130Z8, 02130Z9, 02130ZC, 02130ZF
IABP	5A02110, 5A02210
PVAD	5A0211D, 5A0221D, 02HA3QZ, 02HA3RJ, 02HA3RS, 02HA3RZ
ECMO	5A15223, 5A1522F, 5A1522G, 5A1522H, 5A15A2F, 5A15A2G, 5A15A2H, 5A0920Z
Temporary MCS	5A02110, 5A02210, 5A0211D, 5A0221D, 02HA3QZ, 02HA3RJ, 02HA3RS,<br>02HA3RZ, 5A15223, 5A1522F, 5A1522G, 5A1522H, 5A15A2F, 5A15A2G,<br>5A15A2H, 5A0920Z, 5A02116, 5A02216, 02HA0RJ, 02HA0RS, 02HA0RZ,<br>02HA4QZ, 02HA4RJ, 02HA4RS, 02HA4RZ
LVAD	02HA0QZ
Heart Transplant	02YA0Z0, 02YA0Z1

**Table S2A.** Multivariable Analysis for Predictors of Index Mortality Among Admissions for Heart Failure		
	**Mortality**	
	**Odds Ratio (95% CI)**	**P value**
Female Sex	0.99 (0.96-1.02)	0.404
**Hospitalization type:**		
B vs. A	0.82 (0.78-0.87)	<0.001
C vs. A	0.67 (0.57-0.78)	<0.001
C vs. B	0.81 (0.70-0.94)	0.007
Age, y (per increase in Age by 10 years)	1.67 (1.64-1.70)	<0.001
Hypertension	0.88 (0.85-0.91)	<0.001
Coronary Artery Disease	0.93 (0.90-0.96)	<0.001
Myocardial Infarction	1.72 (1.64-1.81)	<0.001
Atrial Fibrillation/Flutter	1.28 (1.24-1.32)	<0.001
Valvular Disease	0.95 (0.92-0.98)	<0.001
Peripheral Vascular Disease	1.08 (1.04-1.13)	<0.001
Diabetes Mellitus	0.90 (0.87-0.92)	<0.001
CKD, ESRD	1.05 (1.02-1.08)	0.001
Liver Disease	1.43 (1.36-1.50)	<0.001
Coagulopathy	1.38 (1.33-1.44)	<0.001
Chronic Anticoagulant Use	0.76 (0.73-0.78)	<0.001
Malignancy	1.54 (1.47-1.61)	<0.001
Thyroid Disorder	1.04 (1.00-1.07)	0.037
Chronic Lung Disease	1.08 (1.05-1.11)	<0.001
Malnutrition	2.08 (2.00-2.17)	<0.001
Smoking	0.79 (0.77-0.82)	<0.001
Drug Use	0.73 (0.65-0.82)	<0.001
Alcohol Abuse	0.78 (0.71-0.86)	<0.001
Depression	0.94 (0.90-0.98)	0.007
Obesity	0.78 (0.75-0.81)	<0.001
Hyperlipidemia	0.71 (0.69-0.73)	<0.001
Autoimmune Disorder	1.11 (1.04-1.19)	0.002
**Index Presentation**		
Weekend Admission	0.97 (0.94-1.00)	0.046
Elective Admission	1.19 (1.04-1.36)	0.009
**Income by Zip Code**		
1st quartile (reference)		
2nd quartile	1.13 (1.09-1.18)	<0.001
3rd quartile	1.10 (1.06-1.15)	<0.001
4th quartile	1.09 (1.04-1.14)	<0.001
**Primary Payer**		
Medicare (reference)		
Medicaid	0.81 (0.76-0.87)	<0.001
Private Insurance	0.92 (0.87-0.98)	0.006
Self-Pay/No Charge/Other	1.13 (1.03-1.22)	0.006
**Heart Failure Decompensations**		
AKI	2.19 (2.12-2.25)	<0.001
Mechanical Ventilation	9.18 (8.73-9.65)	<0.001
Non-Invasive Ventilation >24 hours	3.49 (3.30-3.69)	<0.001
Cardiogenic Shock	6.20 (5.85-6.57)	<0.001
Cardiac Arrest	22.24 (20.70-23.91)	<0.001
Ventricular Arrythmias	0.85 (0.81-0.90)	<0.001

Hospitalization Type:
Group A - Direct Admission to non-ATC
Group B - Direct Admission to ATC
Group C - Transfer to ATC

Following Abbreviations Apply:
CKD (Chronic Kidney Disease)
ESRD (End-Stage Renal Disease)
AKI (Acute Kidney Injury)
ATC (Advanced Therapy Center)

**Table S2B.** Sensitivity Analysis for Predictors of Mortality Among Index Admission for Heart Failure, with Additional Adjustment for HF-related procedures.		
	**Odds Ratio**	**P value**
Female Sex	0.98 (0.95-1.00)	0.097
Transfer to ATC	0.73 (0.63-0.86)	<0.001
Age, y, per increase in Age by 10 years	1.65 (1.62-1.68)	<0.001
Hypertension	0.89 (0.86-0.92)	<0.001
Coronary Artery Disease	0.95 (0.92-0.98)	<0.001
Myocardial Infarction	1.78 (1.69-1.86)	<0.001
Atrial Fibrillation/Flutter	1.28 (1.24-1.32)	<0.001
Valvular Disease	0.96 (0.94-0.99)	0.013
Peripheral Vascular Disease	1.08 (1.04-1.12)	<0.001
Diabetes Mellitus	0.90 (0.87-0.92)	<0.001
CKD, ESRD	1.04 (1.01-1.08)	0.005
Liver Disease	1.40 (1.33-1.47)	<0.001
Coagulopathy	1.39 (1.34-1.45)	<0.001
Chronic Anticoagulant Use	0.75 (0.72-0.78)	<0.001
Malignancy	1.51 (1.44-1.58)	<0.001
Thyroid Disorder	1.03 (1.00-1.07)	0.046
Chronic Lung Disease	1.07 (1.04-1.10)	<0.001
Malnutrition	2.08 (2.00-2.17)	<0.001
Smoking	0.79 (0.77-0.82)	<0.001
Drug Use	0.70 (0.63-0.79)	<0.001
Alcohol Abuse	0.78 (0.71-0.86)	<0.001
Depression	0.94 (0.90-0.99)	0.009
Obesity	0.78 (0.75-0.80)	<0.001
Hyperlipidemia	0.71 (0.69-0.73)	<0.001
Autoimmune Disorder	1.12 (1.05-1.19)	<0.001
**Index Presentation**		
Weekend Admission	0.97 (0.94-1.00)	0.038
Elective Admission	1.36 (1.19-1.55)	<0.001
**Income by Zip Code**		
1st Quartile (reference)		
2nd Quartile	1.14 (1.10-1.19)	<0.001
3rd Quartile	1.11 (1.07-1.16)	<0.001
4th Quartile	1.09 (1.05-1.15)	<0.001
**Primary Payer**		
Medicare (reference)		
Medicaid	0.79 (0.74-0.85)	<0.001
Private Insurance	0.95 (0.90-1.01)	0.091
Self-Pay/No Charge/Other	1.13 (1.04-1.23)	0.003
**Heart Failure Decompensations**		
AKI	2.19 (2.12-2.26)	<0.001
Mechanical Ventilation	9.54 (9.06-10.07)	<0.001
Non-Invasive Ventilation >24 hours	3.47 (3.28-3.68)	<0.001
Cardiogenic Shock	6.81 (6.40-7.24)	<0.001
Cardiac Arrest	25.63 (23.80-27.59)	<0.001
Ventricular Arrythmias	1.00 (0.95-1.06)	0.985
**Procedures Performed**		
RHC	0.68 (0.63-0.73)	<0.001
PCI	0.35 (0.30-0.41)	<0.001
CABG	0.38 (0.30-0.50)	<0.001
IABP	1.23 (1.05-1.44)	0.010
PVAD	2.53 (2.02-3.16)	<0.001
ECMO	6.07 (4.36-8.45)	<0.001
TAVR	0.41 (0.22-0.75)	0.004
CRT ICD	0.06 (0.05-0.08)	<0.001
LVAD	0.26 (0.19-0.35)	<0.001
Transplant	0.17 (0.12-0.24)	<0.001

Following Abbreviations Apply:
CKD (Chronic Kidney Disease)
ESRD (End-Stage Renal Disease)
AKI (Acute Kidney Injury)
CRT/ICD placement (Cardiac Resynchronization Therapy/Implantable Cardioverter-Defibrillator placement)
PCI (Percutaneous Coronary Intervention);
RHC (Right Heart Catheterization);
CABG (Coronary Artery Bypass Grafting);
TAVR (Transcatheter Aortic Valve Replacement);
Temporary MCS (Mechanical Circulatory Support);
IABP (Intra-Aortic Balloon Pump);
PVAD (Percutaneous Ventricular Assist Device);
ECMO (Extracorporeal Membrane Oxygenation);
LVAD (Left Ventricular Assist Device)
ATC (Advanced Therapy Center).

Table S2C. Multivariable Analysis for Predictors of Transfer to an ATC Among Index Admission for Heart Failure

	**Odds Ratio**	**P value**
Female Sex	1.05 (0.99-1.11)	0.097
Age, y (per increase in Age by 10 years)	0.65 (0.63-0.67)	<0.001
Hypertension	2.91 (2.68-3.17)	<0.001
Coronary Artery Disease	1.55 (1.45-1.66)	<0.001
Myocardial Infarction	3.18 (2.92-3.46)	<0.001
Atrial Fibrillation/Flutter	1.15 (1.08-1.23)	<0.001
Valvular Disease	2.37 (2.22-2.53)	<0.001
Peripheral Vascular Disease	1.32 (1.22-1.42)	<0.001
Diabetes Mellitus	0.99 (0.93-1.04)	0.660
CKD, ESRD	0.84 (0.79-0.89)	<0.001
Liver Disease	1.79 (1.63-1.96)	<0.001
Coagulopathy	1.53 (1.42-1.63)	<0.001
Chronic Anticoagulant Use	1.42 (1.32-1.52)	<0.001
Malignancy	1.41 (1.27-1.57)	<0.001
Thyroid Disorder	1.32 (1.23-1.42)	<0.001
Chronic Lung Disease	1.10 (1.04-1.17)	0.001
Malnutrition	1.89 (1.72-2.09)	<0.001
Smoking	1.48 (1.40-1.57)	<0.001
Drug Use	0.89 (0.78-1.01)	0.068
Alcohol Abuse	1.23 (1.11-1.38)	<0.001
Depression	1.47 (1.36-1.59)	<0.001
Obesity	1.19 (1.12-1.28)	<0.001
Hyperlipidemia	1.33 (1.26-1.42)	<0.001
Autoimmune Disorder	1.49 (1.33-1.68)	<0.001
**Index Presentation**		
Weekend Admission	0.99 (0.94-1.04)	0.654
Elective Admission	1.14 (0.96-1.36)	0.145
**Income by Zip Code**		
1st (reference)		
2nd quartile	1.39 (1.24-1.56)	<0.001
3rd quartile	1.44 (1.27-1.62)	<0.001
4th quartile	1.57 (1.33-1.85)	<0.001
**Primary Payer**		
Medicare (reference)		
Medicaid	0.84 (0.75-0.93)	0.001
Private Insurance	1.30 (1.19-1.41)	<0.001
Self-Pay/No Charge/Other	0.74 (0.64-0.86)	<0.001
**Heart Failure Decompensations**		
AKI	2.13 (1.99-2.29)	<0.001
Mechanical Ventilation	1.57 (1.39-1.76)	<0.001
Non-Invasive Ventilation >24 hours	1.37 (1.17-1.61)	<0.001
Cardiogenic Shock	7.28 (6.57-8.07)	<0.001
Cardiac Arrest	0.91 (0.80-1.04)	0.179
Ventricular Arrythmias	2.47 (2.28-2.68)	<0.001

Following Abbreviations Apply:
CKD (Chronic Kidney Disease)
ESRD (End-Stage Renal Disease)
AKI (Acute Kidney Injury)
ATC (Advanced Therapy Center)

Table S3. Multivariable Analysis for Predictors of Utilization of Right Heart Catheterization and Temporary Mechanical Circulatory Support Among Index Admissions for Heart Failure

	Right Heart Catheterization		Temporary Mechanical Circulatory Support	
	**Odds Ratio (95% CI)**	**P value**	**Odds Ratio (95% CI)**	**P value**
Female Sex	0.87 (0.85-0.89)	<0.001	0.72 (0.68-0.77)	<0.001
**Hospitalization type:**				
B vs. A	3.12 (2.93-3.34)	<0.001	3.27 (2.98-3.58)	<0.001
C vs. A	8.28 (7.49-9.15)	<0.001	5.06 (4.42-5.80)	<0.001
C vs. B	2.65 (2.43-2.89)	<0.001	1.55 (1.37-1.76)	<0.001
Age, y (per increase in Age by 10 years)	0.73 (0.72-0.74)	<0.001	0.74 (0.72-0.76)	<0.001
Hypertension	0.89 (0.86-0.93)	<0.001	1.01 (0.94-1.09)	0.786
Coronary Artery Disease	1.57 (1.54-1.61)	<0.001	1.68 (1.56-1.81)	<0.001
Myocardial Infarction	1.11 (1.06-1.17)	<0.001	2.32 (2.11-2.54)	<0.001
Atrial Fibrillation/Flutter	0.92 (0.90-0.94)	<0.001	1.04 (0.97-1.11)	0.247
Valvular Disease	2.20 (2.15-2.26)	<0.001	1.28 (1.19-1.38)	<0.001
Peripheral Vascular Disease	1.01 (0.98-1.04)	0.617	1.24 (1.13-1.36)	<0.001
Diabetes Mellitus	0.92 (0.90-0.94)	<0.001	1.02 (0.95-1.09)	0.540
CKD, ESRD	0.73 (0.71-0.75)	<0.001	0.76 (0.70-0.81)	<0.001
Liver Disease	1.15 (1.11-1.19)	<0.001	0.67 (0.61-0.74)	<0.001
Coagulopathy	1.13 (1.09-1.17)	<0.001	2.37 (2.19-2.56)	<0.001
Chronic Anticoagulant Use	0.88 (0.85-0.91)	<0.001	0.77 (0.70-0.84)	<0.001
Malignancy	0.75 (0.72-0.79)	<0.001	0.60 (0.51-0.71)	<0.001
Thyroid Disorder	0.98 (0.95-1.01)	0.111	0.82 (0.75-0.90)	<0.001
Chronic Lung Disease	0.91 (0.90-0.93)	<0.001	0.78 (0.72-0.84)	<0.001
Malnutrition	1.09 (1.04-1.13)	<0.001	1.62 (1.47-1.78)	<0.001
Smoking	1.02 (0.99-1.04)	0.171	0.90 (0.84-0.97)	0.004
Drug Use	0.66 (0.63-0.70)	<0.001	0.65 (0.55-0.77)	<0.001
Alcohol Abuse	1.13 (1.08-1.18)	<0.001	0.76 (0.65-0.88)	<0.001
Depression	0.96 (0.93-0.99)	0.014	0.95 (0.85-1.06)	0.341
Obesity	1.12 (1.09-1.15)	<0.001	0.81 (0.74-0.87)	<0.001
Hyperlipidemia	0.98 (0.96-1.00)	0.116	1.01 (0.94-1.08)	0.817
Autoimmune Disorder	1.21 (1.16-1.27)	<0.001	0.79 (0.65-0.96)	0.017
**Index Presentation**				
Weekend Admission	0.82 (0.80-0.84)	<0.001	0.84 (0.78-0.91)	<0.001
Elective Admission	2.67 (2.51-2.84)	<0.001	2.41 (2.14-2.71)	<0.001
**Income by Zip Code**				
1st (reference)				
2nd quartile	1.12 (1.08-1.17)	<0.001	1.16 (1.06-1.26)	<0.001
3rd quartile	1.25 (1.20-1.30)	<0.001	1.07 (0.98-1.16)	0.154
4th quartile	1.40 (1.33-1.47)	<0.001	1.11 (1.01-1.23)	0.252
**Primary Payer**				
Medicare (reference)				
Medicaid	0.93 (0.89-0.96)	<0.001	0.96 (0.86-1.06)	0.385
Private Insurance	1.41 (1.37-1.45)	<0.001	1.53 (1.40-1.68)	<0.001
Self-Pay/No Charge/Other	1.07 (1.02-1.12)	0.010	1.09 (0.94-1.26)	0.252
**Heart Failure Decompensations**				
AKI	1.48 (1.44-1.52)	<0.001	1.57 (1.45-1.70)	<0.001
Mechanical Ventilation	1.10 (1.03-1.18)	0.005	3.21 (2.89-3.56)	<0.001
Non-Invasive Ventilation >24 hours	0.89 (0.84-0.96)	0.001	0.70 (0.53-0.91)	0.007
Cardiogenic Shock	4.89 (4.60-5.18)	<0.001	23.58 (21.32-26.08)	<0.001
Cardiac Arrest	0.67 (0.62-0.73)	<0.001	1.26 (1.12-1.42)	<0.001
Ventricular Arrythmias	1.94 (1.88-2.00)	<0.001	2.00 (1.84-2.18)	<0.001

Hospitalization Type:
- Group A - Direct Admission to non-ATC
- Group B - Direct Admission to ATC
- Group C - Transfer to ATC

Following Abbreviations Apply:
- CKD (Chronic Kidney Disease)
- ESRD (End-Stage Renal Disease)
- AKI (Acute Kidney Injury)
- ATC (Advanced Therapy Center)

Table S4. Sensitivity Analysis of Outcomes and Procedures Performed for Index Heart Failure Admissions, Stratified by Hospital Type based on High-Volume ATCs, Transfer Status, and Patient Sex

Patient Characteristic	Direct to Non-High-Volume ATC (Group A) n = 2,607,860			Direct to High-Volume ATC (Group B) n = 248,867			Transfer to High-Volume ATC (Group C) n = 9,254		
	Male 1,329,116 (51.0)	Female 1,278,745 (49.0)	p-Value	Male 134,810 (54.2)	Female 114,057 (45.8)	p-Value	Male 5,617 (60.7)	Female 3,637 (39.3)	p-Value
Index Mortality	35,059 (2.6)	32,498 (2.5)	<0.001	4,361 (3.2)	3,170 (2.8)	<0.001	719 (12.8)	424 (11.7)	0.315
Length of Stay, days	4 (2 - 6)	4 (2 - 6)	<0.001	5 (3 - 9)	5 (3 - 8)	<0.001	13 (8 - 21)	11 (7 - 18)	<0.001
Estimated Cost, $USD	7,891 (5,027 - 13,062)	7,837 (5,143 - 12,502)	<0.001	10,686 (6,084 - 21,658)	9,816 (5,913 - 18,069)	<0.001	34,755 (18,507 - 71,358)	27,741 (15,609 - 54,062)	<0.001
90-Day Readmission among Survivors	478,450 (37.0)	456,270 (36.6)	<0.001	51,174 (39.2)	43,375 (39.1)	0.746	1,942 (39.7)	1,231 (38.3)	0.415
**Procedures Performed**									
CRT/ICD placement	24,831 (1.9)	11,059 (0.9)	<0.001	4,492 (3.3)	2,152 (1.9)	<0.001	440 (7.8)	180 (5.0)	<0.001
PCI	18,907 (1.4)	11,450 (0.9)	<0.001	2,258 (1.7)	1,234 (1.1)	<0.001	456 (8.1)	253 (7.0)	0.186
RHC	57,813 (4.3)	39,954 (3.1)	<0.001	24,092 (17.9)	13,259 (11.6)	<0.001	3,003 (53.5)	1,593 (43.8)	<0.001
CABG	3,351 (0.3)	1,508 (0.1)	<0.001	685 (0.5)	293 (0.3)	<0.001	156 (2.8)	69 (1.9)	0.043
TAVR	591 (<0.1)	433 (<0.1)	0.003	260 (0.2)	174 (0.2)	0.100	30 (0.5)	<1.0	0.170
Temporary MCS	4,630 (0.3)	2,162 (0.2)	<0.001	4,174 (3.1)	1,351 (1.2)	<0.001	1,087 (19.4)	511 (14.1)	<0.001
IABP	2,369 (0.2)	1,221 (<0.1)	<0.001	2,277 (1.7)	705 (0.6)	<0.001	627 (11.1)	299 (8.2)	0.002
PVAD	1,937 (0.1)	817 (<0.1)	<0.001	786 (0.6)	231 (0.2)	<0.001	362 (6.4)	166 (4.6)	0.022
ECMO	288 (<0.1)	147 (<0.1)	<0.001	801 (0.6)	318 (0.3)	<0.001	291 (5.2)	154 (4.2)	0.205
LVAD	704 (<0.1)	244 (<0.1)	<0.001	2,786 (2.1)	750 (0.7)	<0.001	379 (6.7)	85 (2.3)	<0.001
Heart Transplant	448 (<0.1)	151 (<0.1)	<0.001	3,144 (2.3)	1,042 (0.9)	<0.001	73 (1.3)	29 (0.8)	0.148

Values are presented as number (percentage) for categorical variables and median (interquartile range) for continuous variables.
Following abbreviations apply:
ATC (Advanced Treatment Center);
CRT/ICD placement (Cardiac Resynchronization Therapy/Implantable Cardioverter-Defibrillator placement);
PCI (Percutaneous Coronary Intervention);
RHC (Right Heart Catheterization);
CABG (Coronary Artery Bypass Grafting);
TAVR (Transcatheter Aortic Valve Replacement);
Temporary MCS (Mechanical Circulatory Support);
IABP (Intra-Aortic Balloon Pump);
PVAD (Percutaneous Ventricular Assist Device);
ECMO (Extracorporeal Membrane Oxygenation);
LVAD (Left Ventricular Assist Device).

Table S5. Sensitivity Analysis of the Association of Female Sex with Heart Failure Management and Outcomes In context of High-Volume Advanced Therapy Centers.

**Characteristic**	**Odds Ratio (Multivariate)**	**P value (Multivariate)**
Index Mortality	0.99 (0.96-1.02)	0.385
90-day Readmission	1.03 (1.02-1.04)	<.001
**Procedures Performed**		
RHC	0.87 (0.85-0.89)	<.001
Any MCS	0.73 (0.68-0.78)	<.001
CABG	0.76 (0.70-0.83)	<.001
PCI	0.85 (0.82-0.88)	<.001
TAVR	0.76 (0.66-0.89)	<.001
IABP	0.80 (0.73-0.87)	<.001
PVAD	0.69 (0.62-0.77)	<.001
ECMO	0.88 (0.76-1.03)	0.105
CRT/ICD	0.61 (0.59-0.63)	<.001
LVAD	0.58 (0.51-0.66)	<.001
Transplant	0.62 (0.55-0.69)	<.001

Table S6. Characteristics of Patients with Index Admissions with **Severe Heart Failure**,* Stratified by Patient Sex, Hospital Type, and Transfer

Patient Characteristic	Direct to Non-ATC (Group A) n = 89,843			Direct to ATC (Group B) n = 48,488			Transfer to ATC (Group C) n = 7,825		
	Male	Female	p-Value*	Male	Female	p-Value*	Male	Female	p-Value*
**Demographics**	n=52,957 (58.9)	n=36,886 (41.1)		n=31,383 (64.7)	n=17,105 (35.3)		n=5,234 (66.9)	n=2,591 (33.1)	
**Age (years)**	69 (59-78)	72 (62 - 81)	<0.001[†]	63 (53 - 71)	65 (55 -76)	<0.001[†]	61 (52 - 69)	62 (51-71)	0.013[†]
**Age Group (years)**			<0.001			<0.001			<0.001
<40	1,680 (3.2)	1,028 (2.8)		2,449 (7.8)	1,321 (7.7)		470 (9.0)	342 (13.2)	
40-49	3,329 (6.3)	1,422 (3.9)		3,388 (10.8)	1,286 (7.5)		592 (11.3)	231 (8.9)	
50-59	8,400 (15.9)	4,496 (12.2)		6,762 (21.5)	3,046 (17.8)		1,236 (23.6)	500 (19.3)	
60-69	13,408 (25.3)	8,147 (22.1)		9,268 (29.5)	4,578 (26.8)		1,664 (31.8)	758 (29.3)	
70-79	13,947 (26.3)	10,283 (27.9)		6,161 (19.6)	3,760 (22.0)		962 (18.4)	533 (20.6)	
80+	12,193 (23.0)	11,509 (31.2)		3,355 (10.7)	3,113 (18.2)		311 (5.9)	227 (8.8)	
**Median Household Income by ZIP Code**			<0.001			<0.001			0.142
1st Quartile	18,166 (34.8)	13,159 (36.1)		9,941 (32.1)	6,190 (36.6)		1,308 (25.2)	699 (27.3)	
2nd Quartile	14,208 (27.2)	9,978 (27.4)		7,992 (25.8)	4,269 (25.2)		1,444 (27.8)	752 (29.4)	
3rd Quartile	11,614 (22.3)	8,026 (22.0)		7,232 (23.3)	3,746 (22.1)		1,426 (27.5)	668 (26.1)	
4th Quartile	8,195 (15.7)	5,255 (14.4)		5,833 (18.8)	2,724 (16.1)		1,009 (19.5)	440 (17.2)	
**Comorbidities**									
**Hypertension**	13,976 (26.4)	10,817 (29.3)	<0.001	7,939 (25.3)	4,485 (26.2)	0.150	2,629 (50.2)	1,235 (47.7)	0.134
**Hyperlipidemia**	25,245 (47.7)	16,905 (45.8)	<0.001	13,106 (41.8)	6,865 (40.1)	0.023	2,890 (55.2)	1,295 (50.0)	0.002
**Coronary Artery Disease**	35,357 (66.8)	20,745 (56.2)	<0.001	18,862 (60.1)	8,505 (49.7)	<0.001	3,783 (72.3)	1,645 (63.5)	<0.001
**Prior MI**	6,826 (12.9)	4,862 (13.2)	0.378	3,030 (9.7)	1,911 (11.2)	<0.001	1,099 (21.0)	644 (24.9)	0.006
**Atrial Fibrillation/Flutter**	27,946 (52.8)	17,421 (47.2)	<0.001	16,197 (51.6)	7,587 (44.4)	<0.001	3,084 (58.9)	1,256 (48.5)	<0.001
**Coagulopathy**	10,058 (19.0)	5,839 (15.8)	<0.001	8,369 (26.7)	3,863 (22.6)	<0.001	1,947 (37.2)	875 (33.8)	0.038
**Valve Disease**	15,916 (30.1)	12,360 (33.5)	<0.001	10,245 (32.6)	6,164 (36.0)	<0.001	2,559 (48.9)	1,362 (52.6)	0.055
**Peripheral Vascular Disease**	6,576 (12.4)	4,183 (11.3)	<0.001	3,134 (10.0)	1,563 (9.1)	0.056	789 (15.1)	349 (13.5)	0.258
**Diabetes**	25,037 (47.3)	17,694 (48.0)	0.153	13,190 (42.0)	7,198 (42.1)	0.933	2,324 (44.4)	1,122 (43.3)	0.545
**Chronic Kidney Disease**	33,336 (62.9)	20,749 (56.3)	<0.001	19,999 (63.7)	9,555 (55.9)	<0.001	3,401 (65.0)	1,364 (52.6)	<0.001
**Liver Disease**	7,340 (13.9)	3,212 (8.7)	<0.001	4,873 (15.5)	1,997 (11.7)	<0.001	1,406 (26.9)	541 (20.9)	<0.001
**Long Term Anticoagulant Use**	10,388 (19.6)	6,459 (17.5)	<0.001	6,815 (21.7)	3,151 (18.4)	<0.001	1,471 (28.1)	613 (23.7)	0.009
**Malignancy**	3,100 (5.9)	1,922 (5.2)	0.006	1,463 (4.7)	879 (5.1)	0.144	236 (4.5)	154 (6.0)	0.068
**Thyroid Disorder**	6,394 (12.1)	8,719 (23.6)	<0.001	3,605 (11.5)	3,724 (21.8)	<0.001	800 (15.3)	689 (26.6)	<0.001
**Autoimmune Conditions**	1,030 (1.9)	1,774 (4.8)	<0.001	585 (1.9)	987 (5.8)	<0.001	133 (2.5)	200 (7.7)	<0.001
**Chronic Lung Disease**	20,222 (38.2)	14,955 (40.5)	<0.001	8,123 (25.9)	5,222 (30.5)	<0.001	1,613 (30.8)	1,000 (38.6)	<0.001
**Malnutrition/Weight Loss**	6,440 (12.2)	4,615 (12.5)	0.271	6,136 (19.6)	3,075 (18.0)	0.005	1,268 (24.2)	606 (23.4)	0.598
**Tobacco Use**	22,492 (42.5)	11,765 (31.9)	<0.001	11,259 (35.9)	4,668 (27.3)	<0.001	2,683 (51.3)	1,062 (41.0)	<0.001
**Drug Use**	3,405 (6.4)	1,184 (3.2)	<0.001	1,587 (5.1)	552 (3.2)	<0.001	343 (6.5)	125 (4.8)	0.040
**Alcohol Abuse**	4,538 (8.6)	792 (2.1)	<0.001	1,878 (6.0)	296 (1.7)	<0.001	603 (11.5)	82 (3.1)	<0.001
**Depression**	4,314 (8.1)	4,653 (12.6)	<0.001	2,674 (8.5)	2,211 (12.9)	<0.001	635 (12.1)	530 (20.5)	<0.001
**Obesity**	12,347 (23.3)	10,421 (28.3)	<0.001	6,714 (21.4)	4,541 (26.5)	<0.001	1,388 (26.5)	799 (30.8)	0.009
**Index Presentation**									
**Heart Failure Decompensations**									
Acute Kidney Injury	33,637 (63.5)	20,687 (56.1)	<0.001	21,798 (69.5)	10,590 (61.9)	<0.001	4,344 (83.0)	1,879 (72.5)	<0.001
Mechanical Ventilation/Intubation	29,225 (55.2)	22,939 (62.2)	<0.001	12,050 (38.4)	8,175 (47.8)	<0.001	2,043 (39.0)	1,290 (49.8)	<0.001
Non-Invasive Ventilation >24 hours	2,478 (4.7)	1,960 (5.3)	0.002	825 (2.6)	547 (3.2)	0.008	218 (4.2)	102 (3.9)	0.736
Cardiogenic Shock	25,010 (47.2)	14,229 (38.6)	<0.001	22,894 (72.9)	10,556 (61.7)	<0.001	4,634 (88.5)	2,114 (81.6)	<0.001
Cardiac Arrest	17,651 (33.3)	11,849 (32.1)	0.011	5,959 (19.0)	3,678 (21.5)	<0.001	1,027 (19.6)	546 (21.1)	0.287
Ventricular Arrhythmias	12,534 (23.7)	5,981 (16.2)	<0.001	10,845 (34.6)	4,076 (23.8)	<0.001	2,391 (45.7)	832 (32.1)	<0.001
**Myocardial Infarction**	6,826 (12.9)	4,862 (13.2)	0.378	3,030 (9.7)	1,911 (11.2)	<0.001	1,099 (21.0)	644 (24.9)	0.006
STEMI	766 (1.4)	535 (1.5)	0.967	518 (1.7)	284 (1.7)	0.954	300 (5.7)	170 (6.5)	0.296
**Stroke/TIA**	1,221 (2.3)	971 (2.6)	0.028	1,009 (3.2)	636 (3.7)	0.076	256 (4.9)	107 (4.1)	0.290
**Weekend Admission**	12,392 (23.4)	9,120 (24.7)	0.004	6,002 (19.1)	3,617 (21.1)	<0.001	1,178 (22.5)	621 (24.0)	0.276
**Elective Admission**	2,409 (4.6)	1,634 (4.4)	0.561	4,159 (13.3)	1,718 (10.1)	<0.001	376 (7.2)	159 (6.2)	0.262
**Primary Payer**			<0.001			<0.001			0.025
Medicare	36,353 (68.7)	28,176 (76.5)		17,929 (57.2)	11,202 (65.6)		2,696 (51.6)	1,466 (56.6)	
Medicaid	7,199 (13.6)	4,072 (11.1)		4,589 (14.6)	2,391 (14.0)		809 (15.5)	406 (15.7)	
Private Insurance	6,305 (11.9)	3,527 (9.6)		7,054 (22.5)	2,894 (16.9)		1,474 (28.2)	616 (23.8)	
Self-Pay/No Charge/Other	3,042(5.8)	1,070 (2.9)		1,753 (5.6)	596 (3.5)		249(4.8)	104 (4.0)	
**Disposition Status**			<0.001			<0.001			<0.001
Home/Home Health Care	21,409 (40.4)	13,167 (35.7)		18,643 (59.4)	8,709 (50.9)		3,182 (60.8)	1,340 (51.7)	
Transfer to SNF/ICF/Other	11,175 (21.1)	9,460 (25.6)		4,973 (15.8)	3,483 (20.4)		752 (14.4)	526 (20.3)	
AMA/Unknown/Other	914 (1.7)	298 (0.8)		338 (1.1)	91 (0.5)		39 (0.7)	<20	

Died	19,459 (36.7)	13,961 (37.8)	7,430 (23.7)	4,822 (28.2)	1,261 (24.1)	710 (27.4)

*Severe Heart Failure is defined as presence of cardiogenic shock, cardiac arrest or mechanical ventilation on index admission for heart failure.
** Rao-Scott $\chi 2$ test was used for all statistical tests in comparison to Male patients unless stated otherwise.
† Mann-Whitney-Wilcoxon nonparametric test was used
Values are presented as number (percentage) for categorical values and median (interquartile range) for continuous variables.

Following abbreviations apply:

MI (Myocardial Infarction)
STEMI (ST-elevation Myocardial Infarction)
TIA (Transient Ischemic Attack)
SNF (Skilled Nursing Facility)
ICF (Intermediate Care Facility)
ATC (Advanced Therapy Center)

Table S7. Outcomes and Procedures Performed for Index Admission for Severe Heart Failure,* Stratified by Patient Sex, Hospital Type, and Transfer

Patient Characteristic	Direct to Non-ATC (Group A) n = 89,843			Direct to ATC (Group B) n = 48,488			Transfer to ATC (Group C) n = 7,825		
	Male n=52,957 (58.9)	Female n=36,886 (41.1)	p-Value	Male n=31,383 (64.7)	Female n=17,105 (35.3)	p-Value	Male n=5,234 (66.9)	N=Female n=2,591 (33.1)	p-Value
Index Mortality	19,459 (36.7)	13,961 (37.8)	0.020	7,430 (23.7)	4,822 (28.2)	<0.001	1,261 (24.1)	710 (27.4)	0.054
Length of Stay, days	7.1 (3.4 – 12.9)	6.8 (3.3 – 12.7)	0.010	11.5 (6.0 – 22.2)	10.2 (5.1 – 19.6)	<0.001	16.5 (10.0 – 27.0)	14.5 (8.9 – 24.1)	<0.001
Estimated Cost, $USD	21,160 (11,024 - 41,090)	20,454(11,039 - 38,492)	<0.001	40,679 (18,777 - 112,541)	33,451 (16,682 - 75,662)	<0.001	61,645 (34,071 - 134,696)	52,812 (29,380 - 101,626)	<0.001
90-Day Readmission among Survivors	13,466 (40.2)	8,972 (39.1)	0.078	10,322 (43.1)	5,352 (43.6)	0.539	1,668 (42.0)	764 (40.6)	0.516
**Procedures Performed**									
CRT/ICD placement	2,537 (4.8)	1,291 (3.5)	<0.001	1,752 (5.6)	840 (4.9)	0.059	534 (10.2)	218 (8.4)	0.114
PCI	1,889 (3.6)	1,241 (3.4)	0.256	903 (2.9)	504 (2.9)	0.769	492 (9.4)	199 (7.7)	0.104
RHC	5,535 (10.5)	3,277 (8.9)	<0.001	14,115 (45.0)	5,937 (34.7)	<0.001	3,432 (65.6)	1,405 (54.2)	<0.001
CABG	928 (1.8)	457 (1.2)	<0.001	649 (2.1)	311 (1.8)	0.223	182 (3.5)	80 (3.1)	0.571
TAVR	112 (0.2)	75 (0.2)	0.849	123 (0.4)	85 (0.5)	0.256	<1%	<1%	-
Temporary MCS	2,133 (4.0)	1,160 (3.1)	<0.001	6,229 (19.8)	2,220 (13.0)	<0.001	1,786 (34.1)	695 (26.8)	<0.001
IABP	1,302 (2.5)	738 (2.0)	0.002	3,388 (10.8)	1,169 (6.8)	<0.001	1,043 (19.9)	430 (16.6)	0.011
PVAD	875 (1.7)	415 (1.1)	<0.001	1,285 (4.1)	456 (2.7)	<0.001	598 (11.4)	222 (8.6)	0.016
ECMO	64 (<0.1)	24 (<0.1)	0.063	1,145 (3.6)	493 (2.9)	0.001	437 (8.3)	200 (7.7)	0.571
LVAD**	Not Performed	Not Performed	N/A	3,785 (12.1)	1,126 (6.6)	<0.001	592 (11.3)	150 (5.8)	<0.001
Heart Transplant**	Not Performed	Not Performed	N/A	2,607 (8.3)	891 (5.2)	<0.001	138 (2.6)	40 (1.5)	0.018

*Severe Heart Failure is defined as presence of cardiogenic shock, cardiac arrest or mechanical ventilation on index admission for heart failure.
** LVAD and Heart transplants were not performed in non-ATC centers.
Values are presented as number (percentage) for categorical variables and median (interquartile range) for continuous variables.
Following abbreviations apply:
CRT/ICD placement (Cardiac Resynchronization Therapy/Implantable Cardioverter-Defibrillator placement);
PCI (Percutaneous Coronary Intervention);
RHC (Right Heart Catheterization);
CABG (Coronary Artery Bypass Grafting);
TAVR (Transcatheter Aortic Valve Replacement);
Temporary MCS (Mechanical Circulatory Support);
IABP (Intra-Aortic Balloon Pump);
PVAD (Percutaneous Ventricular Assist Device);
ECMO (Extracorporeal Membrane Oxygenation);
LVAD (Left Ventricular Assist Device)
ATC (Advanced Therapy Center).

Table S8. Multivariable Analysis of The Association of Female Sex with Heart Failure Management and Outcomes among Patients Admitted for Severe Heart Failure.*

Characteristic	Odds Ratio	P value
Index Mortality	0.99 (0.95-1.03)	0.542
90-day Readmission	1.02 (0.98-1.06)	0.294
**Procedures Performed**		
RHC	0.90 (0.85-0.94)	<0.001
Any MCS	0.81 (0.76-0.87)	<0.001
CABG	0.91 (0.80-1.03)	0.142
PCI	0.99 (0.90-1.10)	0.880
TAVR	0.95 (0.72-1.25)	0.695
IABP	0.86 (0.79-0.93)	<0.001
PVAD	0.82 (0.72-0.92)	<0.001
ECMO	0.91 (0.79-1.05)	0.200
CRT/ICD	0.91 (0.80-1.03)	0.129
LVAD	0.66 (0.59-0.75)	<0.001
Transplant	0.74 (0.65-0.84)	<0.001

*Severe Heart Failure is defined as presence of cardiogenic shock, cardiac arrest or mechanical ventilation on index admission for heart failure.

Following abbreviations apply:

CRT/ICD placement (Cardiac Resynchronization Therapy/Implantable Cardioverter-Defibrillator placement);
PCI (Percutaneous Coronary Intervention);
RHC (Right Heart Catheterization);
CABG (Coronary Artery Bypass Grafting);
TAVR (Transcatheter Aortic Valve Replacement);
Temporary MCS (Mechanical Circulatory Support);
IABP (Intra-Aortic Balloon Pump);
PVAD (Percutaneous Ventricular Assist Device);
ECMO (Extracorporeal Membrane Oxygenation);
LVAD (Left Ventricular Assist Device)
ATC (Advanced Therapy Center).

Table S9. Subgroup Analyses of Association of Sex with Procedural Utilization. Multivariable models adjusted for demographic, clinical, and hospital characteristics as described in the primary analysis.

	Overall population		Ischemic Cardiomyopathy/ Myocardial Infarction	
	Odds ratio (95% CI)	P value	Odds ratio (95% CI)	P value
CABG	0.76 (0.70-0.83)	<0.001	0.81 (0.73 - 0.90)	<0.001
PCI	0.84 (0.81-0.87)	<0.001	0.88 (0.84 – 0.92)	<0.001
	Overall population		Systolic Heart Failure	
	Odds ratio (95% CI)	P value	Odds ratio (95% CI)	P value
CRT/ICD	0.61 (0.59-0.63)	<0.001	0.78 (0.76 - 0.81)	<0.001
LVAD	0.58 (0.51-0.67)	<0.001	0.67 (0.59 - 0.77)	<0.001
Transplant	0.63 (0.56-0.70)	<0.001	0.78 (0.69 - 0.88)	<0.001
	Overall population		Cardiogenic Shock	
	Odds ratio (95% CI)	P value	Odds ratio (95% CI)	P value
RHC	0.87 (0.85-0.89)	<0.001	0.84 (0.79 - 0.89)	<0.001
Temporary MCS	0.72 (0.68-0.77)	<0.001	0.87 (0.81 - 0.94)	<0.001
LVAD	0.58 (0.51-0.67)	<0.001	0.68 (0.58 - 0.79)	<0.001
Transplant	0.63 (0.56-0.70)	<0.001	0.74 (0.62 - 0.87)	<0.001

Figure S1. Frequencies of Procedure Utilization, stratified by Patient Sex, Hospitalization Type, and Transfer:

A - Direct Admission to non-ATC

B - Direct Admission to ATC

C - Transfer to ATC

Abbreviations:

RHC: Right Heart Catheterization;

MCS: Mechanical Circulatory Support;

PCI: Percutaneous Coronary Intervention;

MCS: Mechanical Circulatory Support;

CABG: Coronary Artery Bypass Grafting;

PCI: Percutaneous Coronary Intervention;

TAVR: Transcatheter Aortic Valve Replacement

IABP: Intra-Aortic Balloon Pump;

PVAD: Percutaneous Ventricular Assist Device;

ECMO: Extracorporeal Membrane Oxygenation;

CRT/ICD: Cardiac Resynchronization Therapy/Implantable Cardioverter-Defibrillator;

LVAD: Left Ventricular Assist Device

ATC: Advanced Therapy Center

*LVADs and Heart Transplants were not performed in non-ATC centers

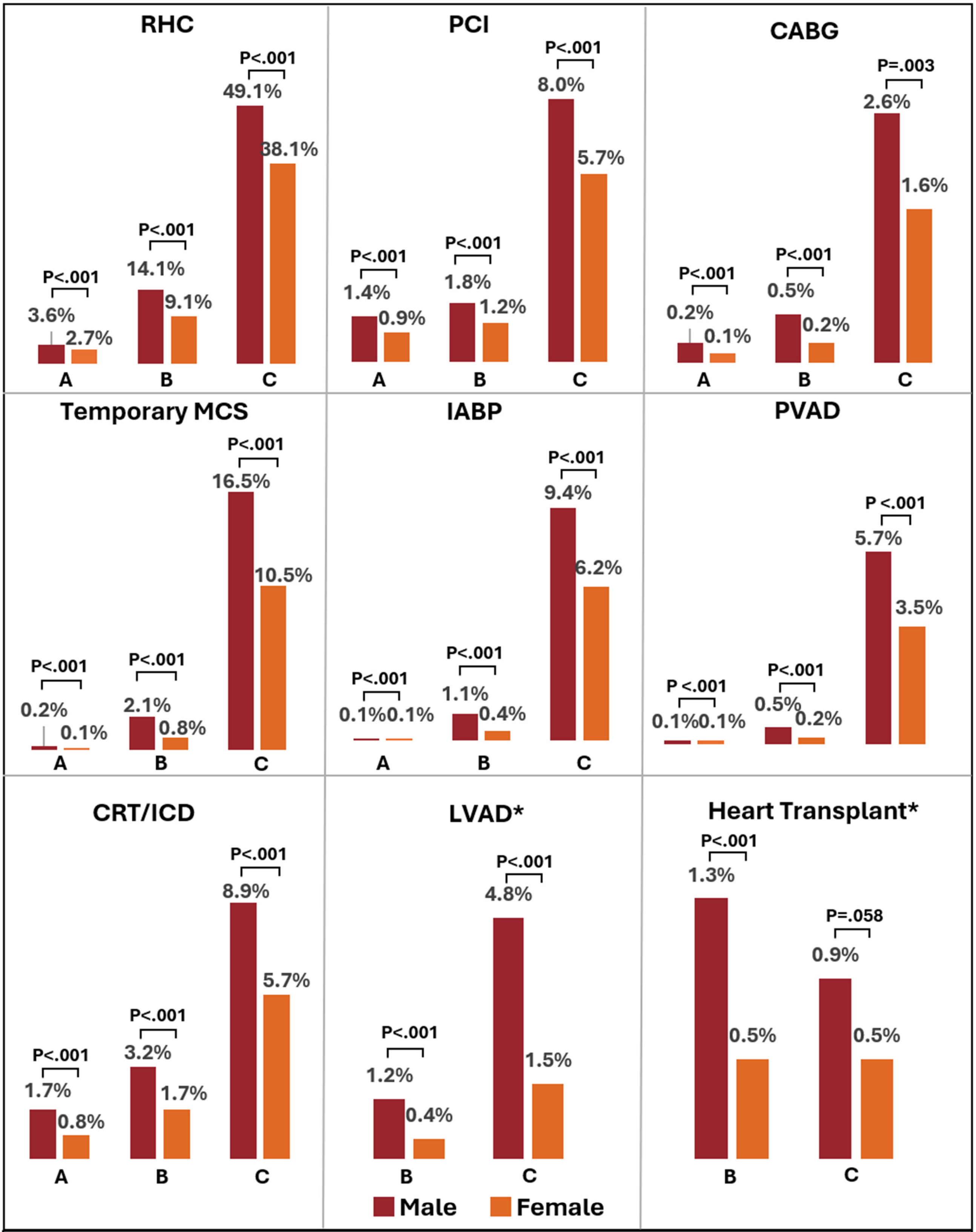

Figure S1. Frequencies of Procedure Utilization, stratified by Patient Sex and Hospitalization Type:
Group A - Direct Admission to non-ATC
Group B - Direct Admission to ATC
Group C - Transfer to ATC
* LVAD and Heart transplants were not performed in non-ATC centers.
